# Molecular signatures of the rediae, cercariae and adult stages in the complex life cycles of parasitic flatworms (Digenea: Psilostomatidae)

**DOI:** 10.1186/s13071-020-04424-4

**Published:** 2020-11-10

**Authors:** Maksim A. Nesterenko, Viktor V. Starunov, Sergei V. Shchenkov, Anna R. Maslova, Sofia A. Denisova, Andrey I. Granovich, Andrey A. Dobrovolskij, Konstantin V. Khalturin

**Affiliations:** 1 Department of Invertebrate Zoology, St-Petersburg State University, Saint Petersburg, 199034 Russia; 2 Zoological Institute, Russian Academy of Sciences, Saint Petersburg, 199034 Russia; 3 Marine Genomics Unit, OIST, 1919-1 Tancha, Onna-son, Kunigami-gun, Okinawa, 904-0495 Japan

**Keywords:** Platyhelminthes, Digenea, Psilostomatidae, Comparative transcriptomics, Complex life cycles

## Abstract

**Background:**

Parasitic flatworms (Trematoda: Digenea) represent one of the most remarkable examples of drastic morphological diversity among the stages within a life cycle. Which genes are responsible for extreme differences in anatomy, physiology, behavior, and ecology among the stages? Here we report a comparative transcriptomic analysis of parthenogenetic and amphimictic generations in two evolutionary informative species of Digenea belonging to the family Psilostomatidae.

**Methods:**

In this study the transcriptomes of rediae, cercariae and adult worm stages of *Psilotrema simillimum* and *Sphaeridiotrema pseudoglobulus*, were sequenced and analyzed. High-quality transcriptomes were generated, and the reference sets of protein-coding genes were used for differential expression analysis in order to identify stage-specific genes. Comparative analysis of gene sets, their expression dynamics and Gene Ontology enrichment analysis were performed for three life stages within each species and between the two species.

**Results:**

Reference transcriptomes for *P. simillimum* and *S. pseudoglobulus* include 21,433 and 46,424 sequences, respectively. Among 14,051 orthologous groups (OGs), 1354 are common and specific for two analyzed psilostomatid species, whereas 13 and 43 OGs were unique for *P. simillimum* and *S. pseudoglobulus*, respectively. In contrast to *P. simillimum*, where more than 60% of analyzed genes were active in the redia, cercaria and adult worm stages, in *S. pseudoglobulus* less than 40% of genes had such a ubiquitous expression pattern. In general, 7805 (36.41%) and 30,622 (65.96%) of genes were preferentially expressed in one of the analyzed stages of *P. simillimum* and *S. pseudoglobulus*, respectively. In both species 12 clusters of co-expressed genes were identified, and more than a half of the genes belonging to the reference sets were included into these clusters. Functional specialization of the life cycle stages was clearly supported by Gene Ontology enrichment analysis.

**Conclusions:**

During the life cycles of the two species studied, most of the genes change their expression levels considerably, consequently the molecular signature of a stage is not only a unique set of expressed genes, but also the specific levels of their expression. Our results indicate unexpectedly high level of plasticity in gene regulation between closely related species. Transcriptomes of *P. simillimum* and *S. pseudoglobulus* provide high quality reference resource for future evolutionary studies and comparative analyses.
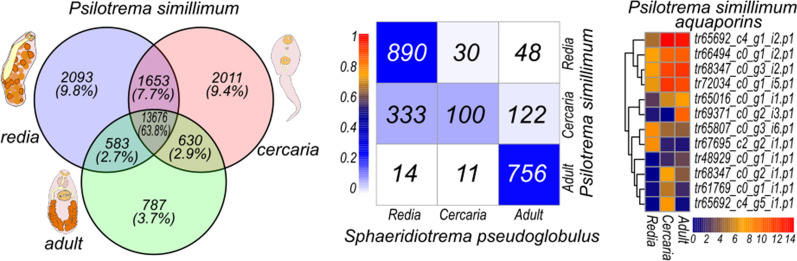

## Background

Complex life cycle with morphologically distinct stages occur in various clades of the animal kingdom [1]. This feature is typical for free-living Cnidaria [2] and Aphidoidea [3] as well as for the parasitic Apicomplexa [4] and Digenea [5]. In contrast to a simple life cycle, which includes only one ontogeny, a complex life cycle is characterized by alternating generations and each of them has its own ontogeny [6].

**Fig. 1:**
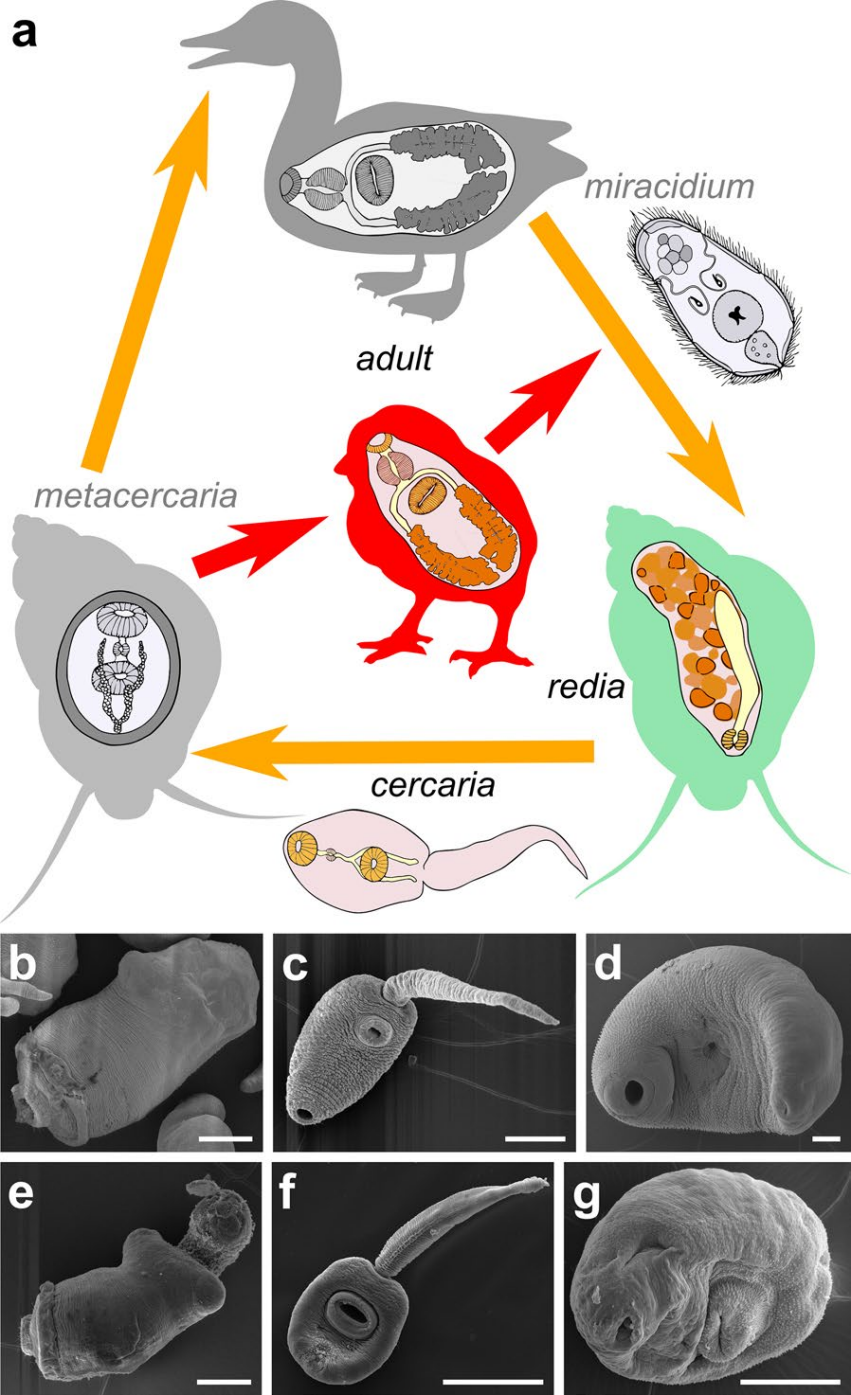
Scheme of the life cycle of the Psilostomatidae and the stages analyzed. **a** Complex life cycle of the Psilostomatidae in nature (yellow arrows) and in the laboratory conditions (red arrows); final host in nature are waterfowl, the first and the second intermediate hosts are molluscs *Bithynia tentaculata*. Chicken was used as final host in laboratory (red silhouette). Redia, metacercaria and adult worm stages are shown inside of their respective hosts. **b**-**g** Scanning electron microscope images of life cycle stages of *Psilotrema simillimum* (**b**-**d**) and *Sphaeridiotrema pseudoglobulus* (**e**-**g**). **b**, **e** Rediae (parasitic stage of the parthenogenetic generation). **c**, **f** Cercariae (free-living larvae of amphimictic generation). **d**, **g** Adult worms of the amphimictic generation. *Scale-bars*: **b**-**e**, **g**, 50 µm; **f**, 100 µm

Digenea is a group of parasitic flatworms that possess one of the most remarkable examples of complex life cycles in the animal kingdom [7]. Throughout life history digeneans usually exploit three hosts (Fig. 1a): the first intermediate host is an invertebrate animal (usually a mollusk), vertebrates or invertebrates may serve as the second intermediate hosts, and vertebrates are used as the definitive hosts [5]. Adult worms are usually hermaphroditic and lay eggs that contain developing miracidia. The aim of a miracidium is to infect the first intermediate host, usually a gastropod mollusk, where it turns into a mother sporocyst. In the so-called ‘redioid’ species a mother sporocyst produces rediae, while in the ‘sporocystoid’ species it gives rise to daughter sporocysts [5]. After several rounds of self-reproduction, individuals of the parthenogenetic generation produce cercariae, free-living larvae of the next amphimictic generation. Cercarial embryos develop within the brood cavity of daughter sporocysts or rediae. Cercariae leave the first intermediate host and spread around in search for the second intermediate host where they turn into metacercariae or the suitable place for turn into adolescariae. The definitive host becomes infected after eating the second intermediate host or adolescariae. In this host, the metacercariae/adolescariae transform into adult worms thereby completing the parasite life cycle (Fig. 1a).

Numerous species of Digenea are of high medical or veterinary importance, e.g. *Paragonimus westermani* Kerbert, 1878 (human lung fluke), *Clonorchis sinensis* Cobbold, 1875 (human liver fluke), *Fasciola hepatica* Linnaeus, 1758 (cattle liver fluke), *Fasciola gigantica* Cobbold, 1855 and, most importantly, schistosomes (blood flukes) [8]. More than a decade ago the genomes of *Schistosoma japonicum* Katsurada, 1904 [9] and *S. mansoni* Sambon, 1907 [10] have been sequenced. Availability of genomic data allowed to study various aspects of digenean biology at the molecular level, including differential gene regulation and epigenetics [11]. Nevertheless, blood flukes possess numerous traits that make them vastly different from the rest of the digeneans. The presence of a schistosomula stage and dioecious amphimictic generation suggest their highly specialized and evolutionary derived state. Over the last ten years, not only the quality of genomes of schistosomes has improved [12], but the genomes of many other species such as *C. sinensis* [13], *Echinostoma caproni* Richard, 1964 [14], *F. hepatica* [14, 15], *Opisthorchis felineus* (Rivolta, 1884) Blanchard, 1895 [16], *O. viverrini* (Poirier, 1886) Stiles & Hassal, 1896 [17], *Schistosoma bovis* Bilharz, 1852 [18], *S. curassoni* Brumpt, 1931 [14], *S. haematobium* Bilharz, 1852 [19], *S. margrebowiei* Le Roux, 1933 [14], *S. mattheei* Lawrence, 1978 [14], *S. rodhaini* Brumpt, 1931 [14] and *Trichobilharzia regenti* Horák, Kolářová & Dvořák, 1998 [14] were sequenced. Despite of the high importance of genomic data, analysis of gene expression changes under different conditions and in the different life cycle stages often remained outside of the main scope in the genome projects. However, comparative analysis of gene activity between various stages is a key to understand the molecular basis of complex life cycle regulation and evolution. Nowadays transcriptomic studies become more abundant and are widely used to study gene expression dynamics during life cycles progression in the free-living [2, 20] and parasitic [21] species.

Comparative transcriptomic analysis of different generations within a life cycle is often impeded by several technical challenges. For example, it is prohibitively difficult to maintain complex life cycles under the laboratory conditions as this requires cultivation of multiple intermediate and definitive hosts. Nevertheless, comparative transcriptomic analysis is indispensable for understanding the origin and evolution of digenean life cycles and could provide new insights for the treatment and prevention of human and animal diseases. Several transcriptomes of digeneans have been sequenced recently [22–35], but in the majority of these studies only adult worms were analyzed [22, 23, 27–31, 33, 35]. Just a few studies analyzed gene expression among different life stages [25, 32, 34] or compared expression dynamics between the same life stages in the different species [23–26, 35].

In this paper we present comparative transcriptomic analysis of the rediae, cercariae and adult worm stages in two digenean species belonging to the family Psilostomatidae, *Psilotrema simillimum* Mühling, 1898 and *Sphaeridiotrema pseudoglobulus* Rudolphi, 1814. Several features make these closely related species a convenient model system. First, they use the same species of prosobranch snail (*Bithynia tentaculata* L.) as the first and second intermediate hosts, which facilitates their maintenance under laboratory conditions. Secondly, shortened uterus of the adult worm is a useful trait for experimental study since it considerably reduces the contamination of adult worm samples by developing miracidia. Free-swimming miracidia of *P. simillimum* and *S. pseudoglobulus* actively infect the first intermediate host and transform into typical mother sporocysts that produce rediae with well-developed gut and an extensive brood cavity which contains numerous developing cercariae. In nature, cercariae of *P. simillimum* leave the first intermediate host to form cysts on the surfaces of water plants or mollusk shells. Resulting metacercariae are eaten by waterfowl and develop into adult worms in their digestive tract. Thus, in the strict sense, *P. simillimum* has a dixenous life cycle. Cercariae of *S. pseudoglobulus* leave the snail host and after a short period of swimming encyst on the shells or under the mantle fold of another *B. tentaculata* snail. The second intermediate host of *Sphaeridiotrema* is usually the same species of a snail and this type of the life cycle can be regarded as a variation of a trixenous one. The adult worms of *S. pseudoglobulus*, similar to *Psilotrema simillimum*, develop in the digestive tract of waterfowl.

The aim of our study was to identify genes with differential expression which possibly contribute to physiological differences between digenean life cycle stages. Another goal was to determine the similarities and differences of the gene expression patterns between *P. simillimum* and *S. pseudoglobulus*.

## Methods

### Animal cultivation

Snail hosts *Bithynia tentaculata* (up to 7000 specimens) were collected from the surface of water plants and stones in the Kristatell’ka river (Peterhof, Russia) and maintained at the Department of Invertebrate Zoology of Saint-Petersburg State University, Saint-Petersburg, Russia. Snails were placed into separate small dishes filled with freshwater for detection of natural infections. Cercariae that emerged from snail hosts were identified according to morphological features [36] under light microscopy. Snails infected with suitable species were selected and placed into separate small dishes with filtered water overnight. Cercariae that emerged from snails were collected into centrifuge tubes and frozen at – 80 °C in IntactRNA™ (Evrogen, Moscow, Russia) reagent according to manufacturer’s protocol.

Rediae were obtained by crushing infected snails in cold physiological solution. Since rediae contain developing cercariae, these two stages cannot be separated. Therefore, after dissection of snail host rediae containing cercariae embyos were immediately placed into IntactRNA™ (Evrogen) reagent and frozen according to the manufacturer’s protocol. In order to obtain metacercariae of *P. simillimum*, shells and water plants were placed with infected snails for about 24 h. Metacercariae of *S. pseudoglobulus* were accumulated directly from snails infected with rediae of the same species.

Metacercariae (up to 200 individuals) in a small amount of water were fed to experimentally incubated domestic chickens before their first natural feeding using a Pasteur pipette. Infected chickens were kept in suitable laboratory conditions for 5 days. Then cervical dislocation of definitive hosts and dissection of their digestive tract were used to collect adult worms. The identification of species was finally confirmed based on adult morphology. Adult worms of *P. simillimum* and *S. pseudoglobulus* were immediately placed into IntactRNA™ (Evrogen) reagent and frozen according to the manufacturer’s protocol.

### RNA isolation and sequencing

The stage-specific pooled samples contained approximately 7 adult worms from 3 chickens, 100 rediae and 300 cercariae from 15 snails respectively for each species were prepared. Prior to RNA isolation, the IntactRNA-fixed samples were rinsed in 0.1 M phosphate-buffered saline (PBS). The total RNA isolation was done using Quick-RNA™ Microprep Kit (R1050; Zymo Research, Irvine, California, USA). For every life cycle stage, two biological replicates were made. The libraries were synthesized using NEBNext Ultra Directional RNA Library Prep Kit for Illumina (E7760; New England BioLabs, Ipswich, Massachusetts, USA). Paired-end sequencing was carried out using Illumina HiSeq 2500 instrument (Illumina, San-Diego, California, USA).

### Reads libraries preparation

The quality of paired end read data was manually assessed using FastQC (v0.11.5). Sequencing error correction was performed by Karect (v1.0) [37] (--celltype=diploid --matchtype=hamming). All libraries were checked for human tissue contamination (Encode: GRCh38, primary assembly) using BBTools packages (v37.02). Additionally, the reference transcriptome of *Bithynia siamensis goniomphalos* [38] and *Gallus gallus* (Encode: *Gallus gallus* v5.0) genome were used for verification of rediae and adult worm libraries host tissue contamination. Sequencing adaptors, low-quality nucleotides and reads with lengths less than 25 nucleotides were removed by Trimmomatic (v0.36) [39] (ILLUMINACLIP:$ADAPTERS:2:30:10:2:TRUE SLIDINGWINDOW:4:20 MAXINFO:50:0.8 MINLEN:25).

### *De novo* reference transcriptomes assembly, redundancy reducing and quality control

For each species RNA-seq data from all prepared libraries were pooled together and used for *de novo* assembly of the reference transcriptomes using Trinity (v2.3.2) [40] with k-mer size and required minimal contig length equal to 25 and 200 nucleotides, respectively. Assembled sequences were renamed by adding in the beginning of sequence IDs three-digit tag of species: ‘Psi’ for *P. simillimum* and ‘Sps’ for *S. pseudogobulus*. Due local database construction rules “DN” in contigs IDs has been replaced to “tr”. Isoform clustering for both transcriptomes was carried out on all assembled contigs with CD-HIT-est (v4.7) [41] and following parameters: sequence identity threshold equal to 95% (-c 0.95), accurate mode (-g 1) and both +/+ and +/− strands alignments (-r 1).

TransRate software (v1.0.1) [42] was used for quality assessment of clustered sequences. Using reads libraries alignments to the contigs, TransRate evaluates the different types of assembling errors (chimeras, structural errors, incomplete assembly and base errors) to produce a diagnostic quality score for each sequence, and these contig scores are integrated to evaluate whole provided transcriptome. Only the contigs which were classified as “good” by TransRate were included into further analysis.

### Genes expression levels quantification and encoded amino acid sequences identification

Salmon (v1.0.1) [43] was used for expression level quantification (-l ISF --discardOrphans --seqBias --gcBias –validateMappings) and only sequences classified as “good” by TransRate were used for index construction. Transcripts-to-gene maps from Trinity were provided for library “tximport” for R language to obtain gene expression values. Scaled TPM (transcripts per million) values were averaged between biological replicates. The genes with averaged expression level below 1 scaledTPM in all analyzed stages were excluded from further analysis.

TransDecoder software (v5.5.0) was used for identification of the amino acid sequences encoded by assembled “good” transcripts in three steps. At the first one, the long open reading frames (ORFs) with length at least 100 amino acids long were found, and the products of its translation were extracted. These amino acid sequences were compared with NCBI non-redundant (DIAMOND BLASTp (v0.9.22.123) (e-value = 1e−3) [44]) and PfamA [45] (HMMscan (v3.1b2)) databases at the second step. At the last step, the comparison results were used as the input for TransDecoder for identification of the likely coding regions and obtaining of the probable set of proteins.

### Reference gene sets identification and sequence annotation

In further analysis we used only sequences that successfully pass two filters: (i) noticeable expression level (i.e. more than 1 scaledTPM in 1 stage); and (ii) protein encoding of a length greater than or equal to 100 amino acids. Only the longest protein and its coding transcript were selected as representatives of each gene. For the gene annotation, their nucleotide and amino acid sequences were compared with available public databases: NCBI nucleotide collection (BLASTn: evalue 1e−3); PfamA (HMMScan); NCBI non-redundant (nr); and SwissProt (DIAMOND BLASTp: --sensitive –evalue 1e−3) databases. In addition, proteins were analyzed using the eggNOG-mapper resource [46] with default parameters for functional annotation.

The presence of the Metazoan single-copy orthologues (Metazoa-odb9) in reference sets of analyzed genes for *P. simillimum* and *S. pseudoglobulus* have been checked with BUSCO (v3.0.1) [47] (e-value = 1e−3, mode = proteins). In order to make corrections for possible gene losses in the Digenea, the metazoan single-copy orthologues searching was also carried out using BUSCO pipeline with same parameters for publicly available proteomes of digeneans. We used the reference proteomes of *Schistosoma mansoni* (UP000008854), *S. haematobium* (UP000054474), *S. japonicum* (UP000311919), *O. viverrini* (UP000054324), *C. sinensis* (UP000286415), *O. felineus* (UP000308267) and *F. hepatica* (UP000230066) from the UniProt database [48] together with the predicted proteomes of *T. regenti* [34], *T. szidati* [26] and *F. gigantica* [25]. For comparison with our results, we excluded sequences with a length less than 100 amino acids from each dataset. All obtained results were assembled in one table with the help of custom script. We call ‛Digenea-specific lossesʼ the orthogroups which had “missing” status in all analyzed datasets.

To estimate differences between *cox*1 mtDNA of *Sphaeridiotrema*, barcode regions of the gene were aligned together with other ones of related species (GenBank: GQ890329.1, GQ890328.1, FJ477222.1, KM538101.1, KM538091.1, KM538104.1, KM538090.1, KM538104.1, MH748721.1, MK982785.1, KY636236.1, KY636202.1, KY636234.1 and MG964028.1). The sequences were automatically aligned using MUSCLE algorithm [49] as implemented in SeaView 4.0 [50]. Bayesian analysis was performed with MrBayes [51, 52] on XSEDE 3.2.7a at CIPRES portal, GTR model with gamma correction for intersite rate variation (8 categories) and the covarion model were used. Trees were run as two separate chains (default heating parameters) for 15 million generations, by which time they had ceased converging (the final average standard deviation of the split frequencies was less than 0.01). The quality of the chains was estimated using built-in MrBayes tools.

### Orthologs identification and analysis

Reference proteomes of *S. mansoni* (UP000008854), *S. haematobium* (UP000054474), *S. japonicum* (UP000311919), *O. viverrini* (UP000054324), *C. sinensis* (UP000286415), *O. felineus* (UP000308267) and *F. hepatica* (UP000230066) from UniProt database [48] together with translated sequences of *T. regenti* [34], *T. szidati* [26], *F. gigantica* [25], *Schmidtea mediterranea* (WormBase Parasites [53]: PRJNA379262) and *Macrostomum lignano* (WormBase Parasites [53]: PRJNA371498) were added to our data for orthogroup reconstruction. Only sequences with length equal to or more than 100 amino acids were used as input for the OrthoFinder (v2.2.6) [54, 55]. Summary statistics were collected with custom scripts written on Python (v3.6) programming language. Species overlap were illustrated using R packages *ggplot2*, *pheatmap* and *RColorBrewer* in RStudio. Overlaps between sets of orthogroups with *P. simillimum*, *S. pseudoglobulus*, ‘redioid’ (*C. sinensis*, *F. gigantica*, *F. hepatica*, *O. felineus* and *O. viverrini*) ‘sporocystoid’ (*S. haematobium*, *S. japonicum*, *S. mansoni*, *T. regenti* and *T. szidati*) and free-living species (*M. lignano* and *S. mediterranea*) were analyzed and visualized with InteractiVenn [56].

For identification of 1-to-1 orthologues for pair of species reciprocal best BLASTp hit (RBBH) search were performed. Pairs of best 1-to-1 matches were found with custom scripts.

### Identification of genes with differential expression in rediae, cercariae and adult worms

A gene was classified as active in the analyzed life cycle stage if its expression level above 1 scaled TPM in this stage. Overlaps between redia, cercaria and adult sets of active genes were analyzed and visualized with Venny (v2.1.0).

We considered a gene to have a differential expression in a stage if ≥ 80% of the total expression of this gene in all analyzed stages were assigned to this one stage of complex life cycle. Genes without such expression dynamics we called “housekeeping” genes. Unlike to popular approaches for differential expression identification, application of described criterion does not require constant expression of most sequences, which is key for comparing different stages of the life cycle, in our opinion. In addition to the two analyzed psilostomatid species, we also used this criterion in a table with expression levels of *F. gigantica* [25]*.* To be able to compare the data from *F. gigantica* with our data, we averaged expression between biological replicates and left only values for redia, cercaria and adult worms.

### Gene Ontology enrichment analysis for sets of differentially expressed genes

Results of differential expression analysis as well as the sequences annotation using eggNOG database were used as the input files for Gene Ontology enrichment analysis carried out with topGO package for R programming language. Analyses were performed on both psilostomatid species and *F. gigantica*. Only terms describing biological processes and molecular functions were analyzed. We used Fisherʼs exact test, and among the results of this analysis (terms with *P*-value < 0.01) extracted only terms in which at least 10 significant genes were included.

### Comparison of gene expression signatures during the life cycle

We used Jaccard similarity scores for measuring the similarity and diversity in RBBH preferential expression and Gene Ontology enrichment analysis results. First, we added information how each sequence in RBBH pairs expressed in the life cycle and measured the similarity between sets of sequences with preferential expression in rediae, cercariae and adult worms. Secondly, we measured similarity between sets of previously identified “enriched” GO-terms, describing biological processes and molecular functions. Visualization of results was performed using packages *ggplot2*, *pheatmap* and *RColorBrewer* in RStudio.

Co-expression clusters for each species separately have been identified using Clust (v1.10.8) [57], using tables with averaged scaledTPM values and default parameters of automatic normalization, cluster tightness of 1.0 and filtering of flat and low expressed genes. Gene Ontology enrichment analysis for co-expressed groups of genes was carried out with *topGO* in R. Only terms describing biological processes and molecular functions were analyzed. We used Fisherʼs exact test and among the results of this analysis (terms with *P*-value < 0.01), extracted only terms in which at least 10 significant genes were included.

Annotation results obtained with eggNOG-mapper were used to analyze the activity of metabolic pathways in redia, cercaria and adult worm stages of *P. simillimum* in terms of number of active enzymes. Pathways which are not active in animal according to Kyoto Encyclopedia of Genes and Genomes (KEGG) or duplicate other were excluded. For visualization we used only pathways which show more than 20% fluctuation between stages in the number of active enzymes involved in a pathway.

### Activity of Wnt and Homeobox genes and preferential expression of cathepsins and aquaporins

Members of *Wnt* signaling cascade, homeobox-containing genes, cathepsins and aquaporins were identified based on the results of HMMer search. Proteins with characteristic Pfam domains were selected and used for further expression analysis and phylogeny reconstruction. Proteins were aligned with MAFFT (v7.130b, with --maxiterate 1000 --localpair options) and poorly aligned areas were removed with TrimAL (v1.2rev.59, with -gappyout option) [58]. Phylogenetic relations among proteins were reconstructed by maximum likelihood (ML) approach using RAxML (v8.2.7) with LG substitution matrix and heterogeneity model GAMMA.

### Immunolabeling and confocal scanning microscopy

Samples were fixed in 4% paraformaldehyde solution in 0.1 M phosphate-buffered saline (PBS) for 8 h at 4 °C, then washed in 0.1 M PBS, incubated in 5% Triton X-100 solution in PBS for 24 h, and blocked in 1% solution of bovine serum albumin in PBS for 6 h. The blocked specimens were incubated in mixture of rabbit anti-5-HT (S5545, diluted 1:1000; Sigma-Aldrich, St. Louis, USA) and rabbit anti-FMRFamide antibodies (AB15348, diluted 1:1000; EMD Millipore, Burlington, Massachusetts, USA). Rediae were incubated in primary antibody for 24 h, then washed in PBS with 0.1% Triton X-100 and incubated in the secondary anti-rabbit CF488 antibody (SAB4600044, diluted 1:1000; Sigma-Aldrich) for 8 h. After antibody incubations samples were washed in PBS, and then incubated in DAPI staining solution (MBD0015, diluted 1:1000; Sigma-Aldrich) for 5 min, washed in PBS and mounted in glycerol. The specimens were examined under Leica TCS SP5 MP confocal laser scanning microscope (Leica Microsystems, Wetzlar, Germany) and analyzed using Fiji software [59].

### Scanning electron microscopy

For scanning electron microscopy, samples were fixed in a cold 2.5% glutaraldehyde solution in 0.05 M sodium cacodylate buffer (SCB) and post-fixed with 2% osmium tetraoxide solution in 0.05 M SCB for 1 h at the room temperature. Samples were dehydrated through a graded ethanol series and transferred through an ethanol-acetone mixture to pure acetone. Then samples were critical point dried, sputtered with platinum, and examined using Tescan MIRA3 LMU scanning electron microscope (Tescan, Brno, Czech Republic).

## Results

### Sequencing and assembly of transcriptomes

Transcriptome analysis in *P. simillimum* and *S. pseudoglobulus* were carried out for three life cycle stages: redia, cercaria and adult worm (Fig. 1b–g). For each stage two biological replicates were sequenced with at least 23.3 million read pairs per sample (Additional file 1: Table S1). Raw Illumina reads were screened for low quality regions and potential contaminations with the sequences from *B. tentaculata* (intermediate host), chicken (experimental final host) (Fig. 1a) and human. Contaminations with host-derived sequences did not exceed 5.4% of the total number of reads in each library (Additional file 1: Table S1). More than 93% of reads remained in all the libraries after the removal of potential host-derived sequences, illumina adapters and poor-quality reads (Additional file 1: Table S1).

Transcriptomes were assembled *de novo* using Trinity (v2.3.2) [40] which resulted in 247,252 and 288,060 contigs for *P. simillimum* and *S. pseudoglobulus*, respectively. In order to reduce redundancy, sequences were clustered with CD-HIT-EST [41] and their assembly quality has been checked with TransRate [42]. More than 95% of the sequences were well-assembled according to TransRate scores (Additional file 2: Table S2). After removal of the sequences with poor TransRate scores the reference transcriptomes for *Psilotrema* and *Sphaeridiotrema* contained 180,786 and 201,608 contigs, respectively (Additional file 2: Table S2).

### Reference gene sets identification

Expression level quantification was carried out with Salmon (v1.0.1) [43] by mapping of trimmed reads against the reference transcriptomes. Mapping rate varied from 78% to 90% (Additional file 3: Table S3). Expression values of splice variants (termed “isoforms” in Trinity) were summed up to obtain gene expression values and all the genes where expression level was low (< 1TPM) in all the stages were excluded from further analysis. As a result, we obtained the datasets filtered based on the expression levels with 144,535 and 150,816 contigs for *P. simillimum* and *S. pseudoglobulus*, respectively.

In further analysis we focused on protein-coding genes only. According to TransDecoder (v5.5.0) predictions, 32,986 and 69,006 sequences in *P. simillimum* and *S. pseudoglobulus* transcriptomes, respectively, encoded proteins with lengths equal or longer than 100 amino acids. After filtering by gene expression level (≥ 1 transcript per million (TPM) in at least one stage) and protein lengths (≥ 100 aa) we obtained the reference sets of sequences for each species: 21,433 genes for *P. simillimum* (Additional file 2: Table S2) and 46,424 genes for *S. pseudoglobulus* (Additional file 2: Table S2). For each gene, the longest protein encoded among all of its splice variants was selected as a representative sequence. All thereby selected nucleotide and amino acid sequences were searched against NCBI nucleotide (NCBInt) and protein non-redundant (NCBInr), SwissProt, PfamA and eggNOG databases. In *P. simillimum* and *S. pseudoglobulus*, 74% (15,865) and 82.5% (38,316) of sequences were annotated with the help of at least one database, respectively.

**Fig. 2:**
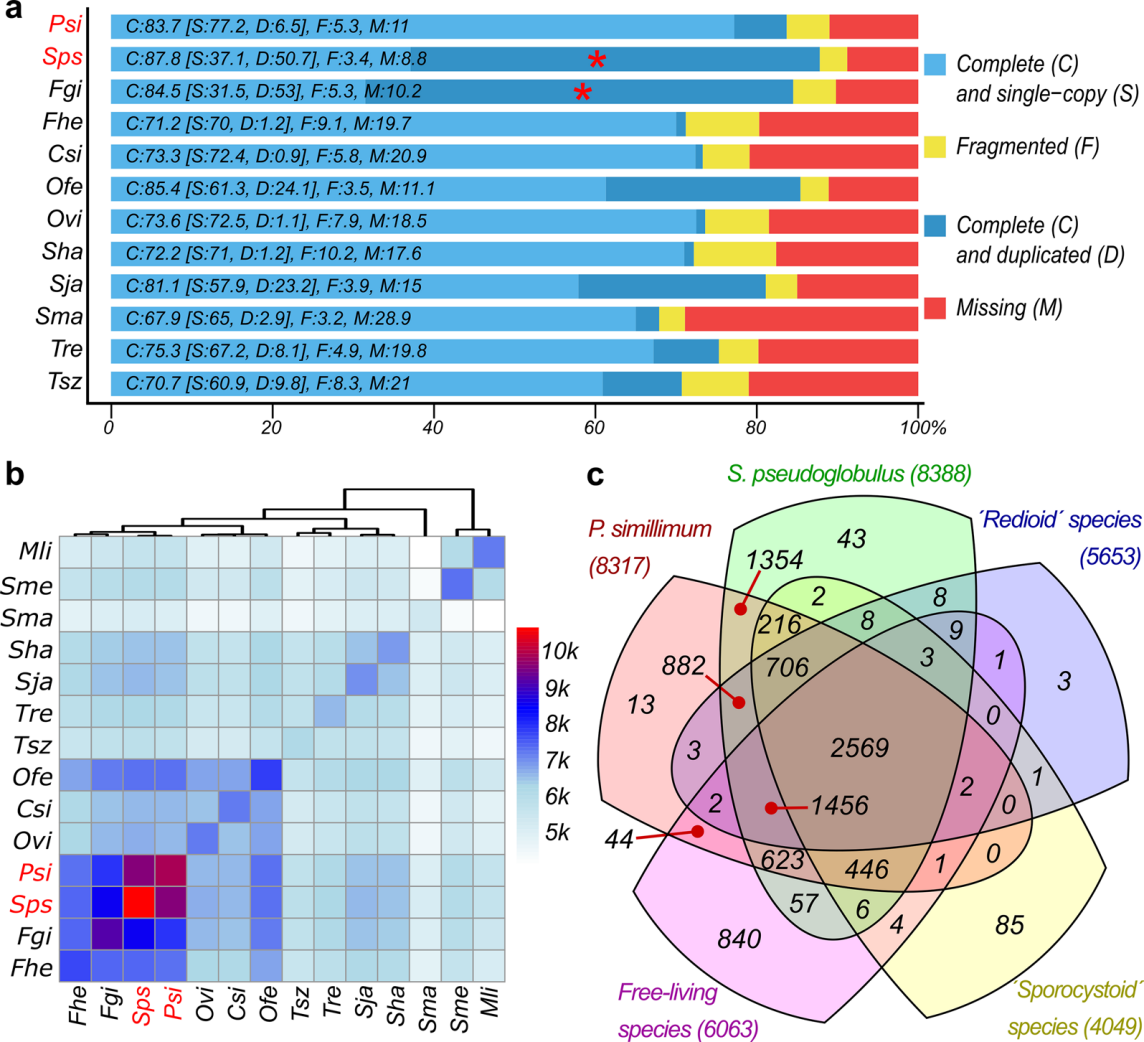
Gene sets in flatworms. **a** BUSCO values for the transcriptome assemblies of *Psilotrema simillimum* (Psi) and *Sphaeridiotrema pseudoglobulus* (Sps) compared to that in previously published datasets of parasitic flatworms. High proportion of duplicated genes in *S. pseudoglobulus* and *F. gigantica* is marked by red asterisk. *Abbreviations*: Psi, *Psilotrema simillimum*; Sps, *Sphaeridiotrema pseudoglobulus*; Fgi, *Fasciola gigantica*; Fhe, *Fasciola hepatica*; Csi, *Clonorchis sinensis*; Ofe, *Opisthorchis felineus*; Ovi, *Opisthorchis viverrini*; Sha, *Schistosoma haematobium*; Sja, *Schistosoma japonicum*; Sma, *Schistosoma mansoni*; Tre, *Trichobilharzia regenti*; Tsz, *Trichobilharzia szidati*. **b** Clustering of shared orthologous proteins among free-living and parasitic flatworms. Based on the similarity of protein sets, four clusters are evident. Abbreviated genus/species names are shown at the bottom and on the left-hand side. *Abbreviations*: Mli, *Macrostomum lignano*; Sme, *Schmidtea mediterranea*; Sma, *Schistosoma mansoni*; Sha, *Schistosoma haematobium*; Sja, *Schistosoma japonicum*; Tre, *Trichobilharzia regenti*; Tsz, *Trichobilharzia szidati*; Ofe, *Opisthorchis felineus*; Csi, *Clonorchis sinensis*; Ovi, *Opisthorchis viverrini*; Psi, *Psilotrema simillimum*; Sps, *Sphaeridiotrema pseudoglobulus*; Fgi, *Fasciola gigantica*; Fhe, *Fasciola hepatica*. The color key shows the number of shared orthogroups among the species. **c** Venn diagram shows the numbers of orthologous groups that are shared by all flatworms (2569), specific for free-living flatworms (*M. lignano* and *S. mediterranea*), shared by all 'sporocystoid' (*S. mansoni*, *S. haematobium*, *S. japonicum*, *T. regenti*, and *T. szidati*) or 'redioid' (*C. sinensis*, *O. viverrini*, *O. felineus*, *F. hepatica* and *F. gigantica*) species, and those that are specific for *P. simillimum* (13) and *S. pseudoglobulus* (43)

Completeness of the reference set of sequences for each species was estimated by comparison with BUSCO Metazoan orthologues database V9. As shown in Fig. 2a, BUSCO values for *P. simillimum* (C + F: 89%) and *S. pseudoglobulus* (C + F: 91.2%) reference proteins sets indicate high level of completeness and are compatible or better than the published datasets from other species of Digenea. Proportion of duplicated genes in *S. pseudoglobulus* (D: 50.7%), similar to that in *F. gigantica* (D: 53%), is unusually high and is on average 8-fold higher than that in *P. simillimum* (D: 6.5%). Proportion of the missing orthologues (11% in *P. simillimum* and 8.8% in *S. pseudoglobulus*) is consistent with that in other analyzed digenean species (Fig. 2a, Additional file 4: Table S4) and most probably represents lineage-specific gene losses. In support of this view, 57 orthologues from BUSCO Metazoa-odb9 dataset were absent in all analyzed Platyhelminthes (Additional file 5: Table S5).

The number of genes in *S. pseudoglobulus* reference dataset was approximately 2-fold larger than that in *P. simillimum.* According to BUSCO analysis the former dataset had unusually high level of duplicated genes (50.7%) (Fig. 2a). Taking into consideration that CD-HIT-EST with 95% similarity cut-off did not remove this redundancy that might indicate that *S. pseudoglobulus* samples contained a mixture of several cryptic species that could not be distinguished based on their morphology. Indeed, it is known that the specimens from waterfowl had been historically identified as a single species (*Sphaeridiotrema globulus* Rudolphi, 1814), but later a new species of *S. pseudoglobulus* McLaughlin, Scott & Huffman, 1993 has been described [60]. Morphological identification of these two species is extremely complicated even by using adult worms and impossible when using their larval stages [60]. Taking this into consideration we screened the transcriptome of *S. pseudoglobulus* for the sequences of mitochondrial cytochrome *c* oxidase subunit 1 gene (*cox*1) and analyzed them together with other available *cox*1 sequences of the closely related digenean species. As a result, two haplotypes of the *cox*1 gene were identified which indicates the presence of two cryptic species among the sampled specimens of *S. pseudoglobulus*. One of the *cox*1 sequences (GenBank: MT934401) is almost identical to the sequences of *S. pseudoglobulus* published previously [60, 61] while another one (GenBank: MT934400) differs from any *cox*1 sequence on GenBank (Additional file 6: Figure S1). Thus, the transcriptome of *S. pseudoglobulus* contains the sequences from the second, not yet described cryptic species closely related to *S. pseudoglobulus.* It is not possible to distinguish these species based on morphological criteria. Unexpected presence of a cryptic species complicates direct comparison of gene expression between *P. simillimum* and *S. pseudoglobulus* as in the latter transcriptomes a large proportion of sequences is represented by two orthologues that stem from the closely related, but still different cryptic species.

### Orthologous groups and gene sets in flatworms

In order to obtain a global view of the gene sets present in *P. simillimum* and *S. pseudoglobulus* we identified their orthologues in 10 species of Digenea (*C. sinensis*, *F. gigantica*, *F. hepatica*, *O. felineus*, *O. viverrini*, *S. haematobium*, *S. japonicum*, *S. mansoni*, *T. regenti* and *T. szidati*) and two species of free-living flatworms (*Macrostomum lignano* and *Schmidtea mediterranea*) (Additional file 7: Tables S6.1, S6.4). In total OrthoFinder assigned 258,413 proteins (83.0% of total) to 14,051 orthogroups (Additional file 7: Tables S6.2, S6.4). Fifty percent of all proteins were in orthogroups with 25 or more genes (G50 = 25) and were contained in the largest 3587 orthogroups (Additional file 7: Table S6.2). There were 2569 orthogroups with the proteins from all species present and none of them consisted entirely of single-copy genes (Additional file 7: Table S6.2). Detailed statistics per species are shown in Additional file 7.

As shown in Fig. 2b, in terms of gene sets, *P. simillimum* and *S. pseudoglobulus* grouped together with *F. gigantica* and *F. hepatica*. About 10,000 orthologous groups (OG) were shared between both psilostomatid species of and about 7000–8000 OGs between psilostomatids and fasciolids. The elevated number of OGs, caused probably by the presence of the cryptic species, is clearly observed in *S. pseudoglobulus*. Overall, four clusters of species that correspond well to their phylogenetic relationships were clearly detectable. The first cluster united psilostomatids and fasciolids, the second cluster contained three species of the Opisthorchiidae (*O. felineus*, *O. viverrini* and *C. sinensis*), and the third cluster united schistosomatids *S. mansoni*, *S. japonicum*, *S. haematobium*, *T. regenti* and *T. szidati*. As expected, free-living flatworms formed a distinct outgroup.

All groups of parasitic worms differ in morphological and physiological adaptations which should be reflected in their corresponding gene sets. Given species-specific differences, we considered only orthogroups that include all species united by a common trait: free-living lifestyle, redia or daughter sporocyst in complex life cycle. As shown in Fig. 2c, 2569 OGs were shared among *P. simillimum*, *S. pseudoglobulus* and other ‘redioid’ (*C. sinensis*, *O. felineus*, *O. viverrini*, *F. hepatica* and *F. gigantica*), ‘sporocystoid’ (*S. mansoni*, *S. japonicum*, *S. haematobium*, *T. regenti* and *T. szidati*) and free-living (*M. lignano* and *S. mediterranea*) species representing the core set of genes in the flatworms analyzed. Detailed analysis of species composition of orthogroups showed that 840 orthogroups include proteins that are present exclusively in the free-living species, *M. lignano* and *S. mediterranea* (Fig. 2c). Three orthologous groups included only *C. sinensis*, *F. gigantica*, *F. hepatica*, *O. felineus* and *O. viverrini* (Fig. 2c)*.* At the same time, proteins that are present only in the species with daughter sporocysts (*S. mansoni*, *S. haematobium*, *S. japonicum*, *T. regenti* and *T. szidati*) were included in 85 unique orthogroups (Fig. 2c). A total of 706 orthogroups contained only digenean species analyzed (Fig. 2c); these groups can be classified as “digenean-specific”. There was 4-fold difference in the number of orthogroups common for both the Psilostomatidae and all ‘redioid’ (*n* = 882), and all ‘sporocystoid’ (*n* = 216) species under consideration (Fig. 2c). The differences found are most likely associated with differences in the biology of ‘redioid’ and ‘sporocystoid’ species.

As shown in Fig. 2c, 1354 orthogroups were common and specific for the two psilostomatid species analyzed. Interestingly, 13 OGs and 43 OGs were unique for *P. simillimum* and *S. pseudoglobulus*, respectively (Fig. 2c, Additional file 8: Table S7). In *P. simillimum*, these OGs include 34 sequences, for example, F-box/WD repeat-containing protein 10 and Netrin receptor UNC5B (see Additional file 8: Table S7). A total of 435 genes were included into OGs, specific for *S. pseudoglobulus*, and included, for example, cubilin-like protein and ABC transporter (Additional file 8: Table S7). At the same time, 52.9% (18) and 70.5% (307) of proteins in *Psilotrema-* and *Sphaeridiotrema-*specific orthogroups respectively, do not have any annotation (Additional file 8: Table S7). Similarly, 50.1% (1826) of sequences belonging to 1354 OGs that are exclusively present in *P. simillimum* and *S. pseudoglobulus* (Fig. 2c) did not have matches in NCBI nr, SwissProt, PfamA, and eggNOG databases (Additional file 8: Table S7).

### Differential expression in the rediae, cercariae and adult worm stages

**Fig. 3:**
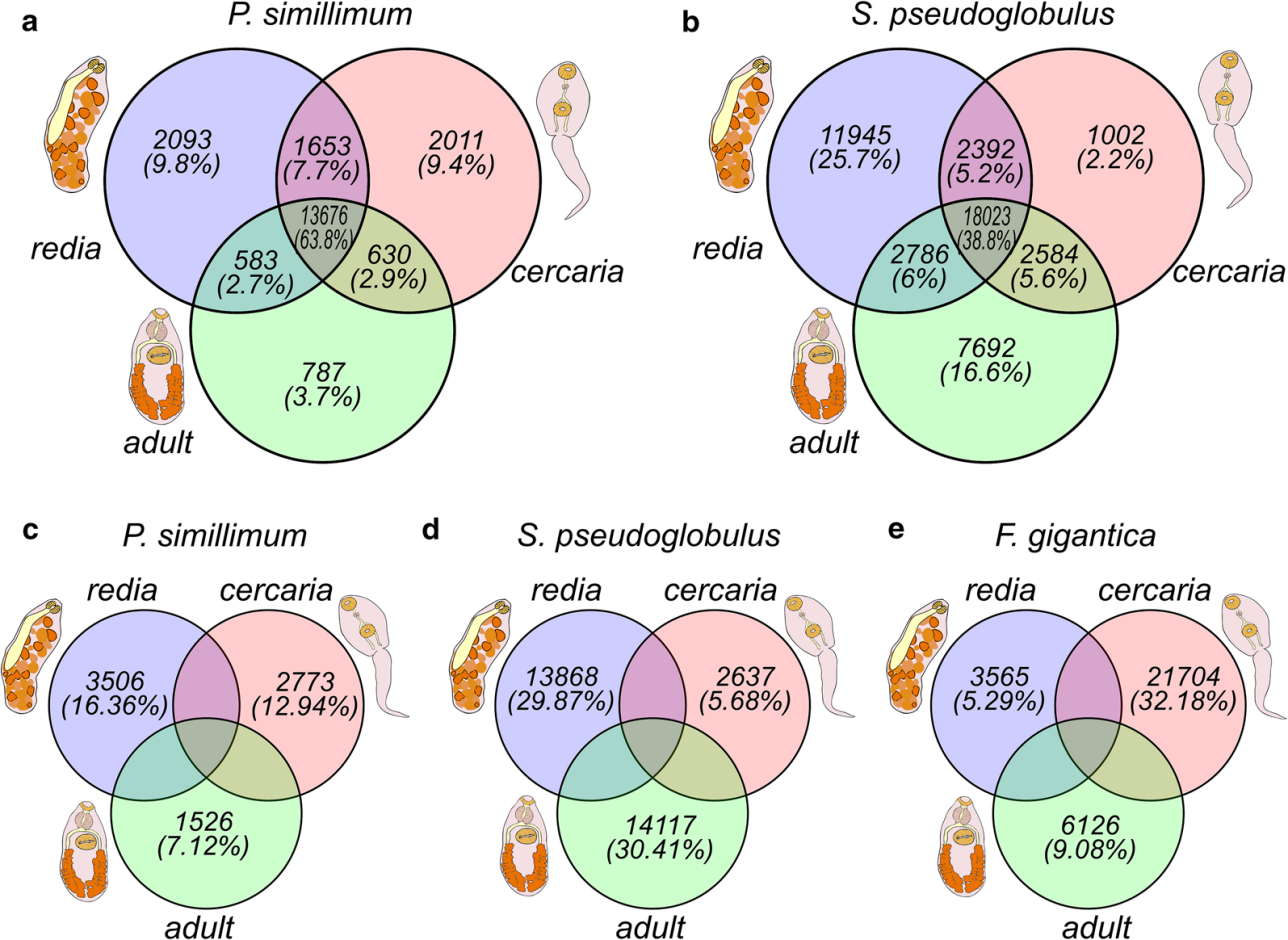
Classification of genes according to their expression in redia, cercaria and adult worm stages. Only genes those expression level exceeds 1 TPM in at least one stage of the life cycle were analyzed. Venn diagram shows the number of active genes in *Psilotrema simillimum* (**a**) and *Sphaeridiotrema pseudoglobulus* (**b**) and the number of differentially expressed genes in *P. simillimum* (**c**), *S. pseudoglobulus* (**d**) and *Fasciola gigantica* (**e**)

Each stage of a life cycle is characterized by structures and functions which are not present in the other stages. Thus, different sets of genes must be activated to allow these differences. Which proportion of genes in the genome are expressed in the three analyzed stages and how many of them show differential expression, i.e. might be responsible for stage-specific traits? In order to answer the first question, we found sequences with expression level equal to or above 1 TPM in rediae, cercariae and adult worms, and compared these sets (Fig. 3a, b). The proportion of sequences that are active in all analyzed stages varied significantly between species: 63.8% (13,676) of genes in *P. simillimum* and 38.8% (18,023) of genes in *S. pseudoglobulus* (Fig. 3a, b). In total, only 22.9% (9.8% + 9.4% + 3.7%) of *P. simillimum* genes showed expression levels ≥ 1 TPM in only one of the stages, whereas in *S. pseudoglobulus* such sequences constituted 44.5% (25.7% + 2.2% + 16.6%) of the reference gene set (Fig. 3a, b). This approach allows to identify genes being switched “on” and “off”, but not those which are characterized by preferential expression in one of the stages, i.e. exhibit stage-specific expression dynamics.

We considered a gene to have a stage-specific expression, if ≥ 80% of the total expression of this gene was restricted to one stage of the life cycle. Normalized TPM values were used as an expression level measure. According to this strict criterion, 16.36% (3506), 12.94% (2773) and 7.12% (1526) of genes in *P. simillimum* were differentially expressed in the redia, cercaria and adult worm stage, respectively (Fig. 3c). These results contrast with *S. pseudoglobulus*, where 29.87% (13868), 5.68% (2637) and 30.41% (14117) of genes were differently expressed in the redia, cercaria and adult worm stage, respectively (Fig. 3d). Application of the same 80% expression criterion to the recently published dataset of *F. gigantica* [25] showed that 5.29% (3565) of sequences had differential expression in the redia, 32.18% (21704) in the cercaria, and 9.08% (6126) in adult worm stage (Fig. 3e). Thus, the number of stage-specific genes in three analyzed ‘redioid’ species differs considerably. However, in *S. pseudoglobulus* the absolute numbers of genes with stage-specific expression were obscured by the presence of two cryptic species which most probably caused elevated counts of redia-specific (13,868, 29.87%) and adult-specific (14,117, 30.41%) genes. Since the morphological differences were observed only among cercariae, it was possible to avoid contamination on this life cycle stage.

### Gene Ontology enrichment analysis for the sets of differentially expressed genes

Gene Ontology (GO) enrichment analysis was used to obtain a global view of biological processes executed by the stage-specific genes. Comparison between *P. simillimum* life cycle stages revealed that the largest number of “enriched” biological processes, 333 GO terms, was assigned to redia stage. These GO terms generally describe immune response, developmental processes and, probably, parthenogenesis (“Single fertilization”, “Female pregnancy”, “Fertilization”) (Additional file 9: Table S8.1). Sequences with specific expression in *P. simillimum* cercaria are involved in 223 biological processes comprising “Skeletal muscle contraction”, “Regulation of skeletal muscle adaptation”, “Transition between fast and slow fiber”, “Relaxation of muscle” and many other (Additional file 9: Table S8.2). In adult worms, among 47 “enriched” terms several are associated with cell motility (“Cilium organization”, “Cilium assembly”, “Cilium movement”, “Axonemal dynein complex assembly”, “Cilium-dependent cell motility”, “Cell motility”), fertilization (“Sperm motility”, “Flagellated sperm motility”, “Spermatid development” and “Spermatid differentiation”) and development (“Determination of left/right symmetry”, “Specification of symmetry”, “Determination of bilateral symmetry”, “Pattern specification process”) (Additional file 9: Table S8.3).

Among 263 enriched terms for the redia stage of *S. pseudoglobulus*, many are associated with cell proliferation (Additional file 9: Table S8.4), for example, “Chromosome organization”, “DNA replication”, “Cell cycle DNA replication”, “Cell cycle phase transition”, “Mitotic nuclear division”, etc. High diversity of biological processes was found in the set of 347 enriched GO-terms for the cercaria of *S. pseudoglobulus*. Some of them are connected with homeostasis, cellular transport, signaling pathways (“Torso signaling pathway”, “BMP signaling pathway”, “Neuropeptide signaling pathway”, etc.), muscular activity (“Muscle contraction”, “Relaxation of muscle” and other), behavior (“Larval locomotory behavior”, “Circadian behavior”, “Rhythmic behavior”, “Circadian sleep/wake cycle”, etc.) and oocytes (“Oocyte dorsal/ventral axis specification”, “Oocyte axis specification”, “Oocyte construction”, “Oocyte development”) (Additional file 9: Table S8.5). Similar to that in *P. simillimum*, in the adult worms of *S. pseudoglobulus*, large proportion of enriched processes can be grouped into two categories: locomotion (“Cilium movement”, “Sperm motility”, “Locomotion”, “Adult behavior”, “Feeding behavior”, “ Chemosensory behavior”, “Taxis”, etc.) and development (“Determination of left/right symmetry”, “Autonomic nervous system development”, “Forebrain morphogenesis”, “Perineurial glial growth”, “Smooth muscle tissue development”, etc.) (Additional file 9: Table S8.6).

According to GO-enrichment analysis, in the redia stage of *F. gigantica*, 188 enriched terms included numerous biological processes associated with cell cycle: “DNA replication”, “Chromosome condensation”, “Cell cycle phase transition”, “Mitotic cell cycle phase transition”, and others (Additional file 9: Table S8.7). Genes with cercaria-specific expression participate in 1555 biological processes responsible for nervous system functions, signaling, development and behavior (Additional file 9: Table S8.8). 186 GO-terms were classified as enriched for adult worms of *F. gigantica*, and part of them, similar to that in the Psilostomatidae, describe cell motility (“Cilium organization”, “Cilium movement”, “Sperm motility”, “Germ cell migration”, “Cell motility”, etc.) and formation of gametes (“Oocyte fate determination”, “Chromosome organization involved in meiotic cell cycle”, “Meiotic chromosome segregation”, “Germarium-derived oocyte differentiation”, “Oocyte dorsal/ventral axis specification”, “Meiosis I”, “Oocyte microtubule cytoskeleton organization”) (Additional file 9: Table S8.9).

Enriched GO-terms describing molecular functions for each analyzed stage are listed in Additional file 10: Table S9.

### Comparison of gene expression signatures during the life cycle

**Fig. 4:**
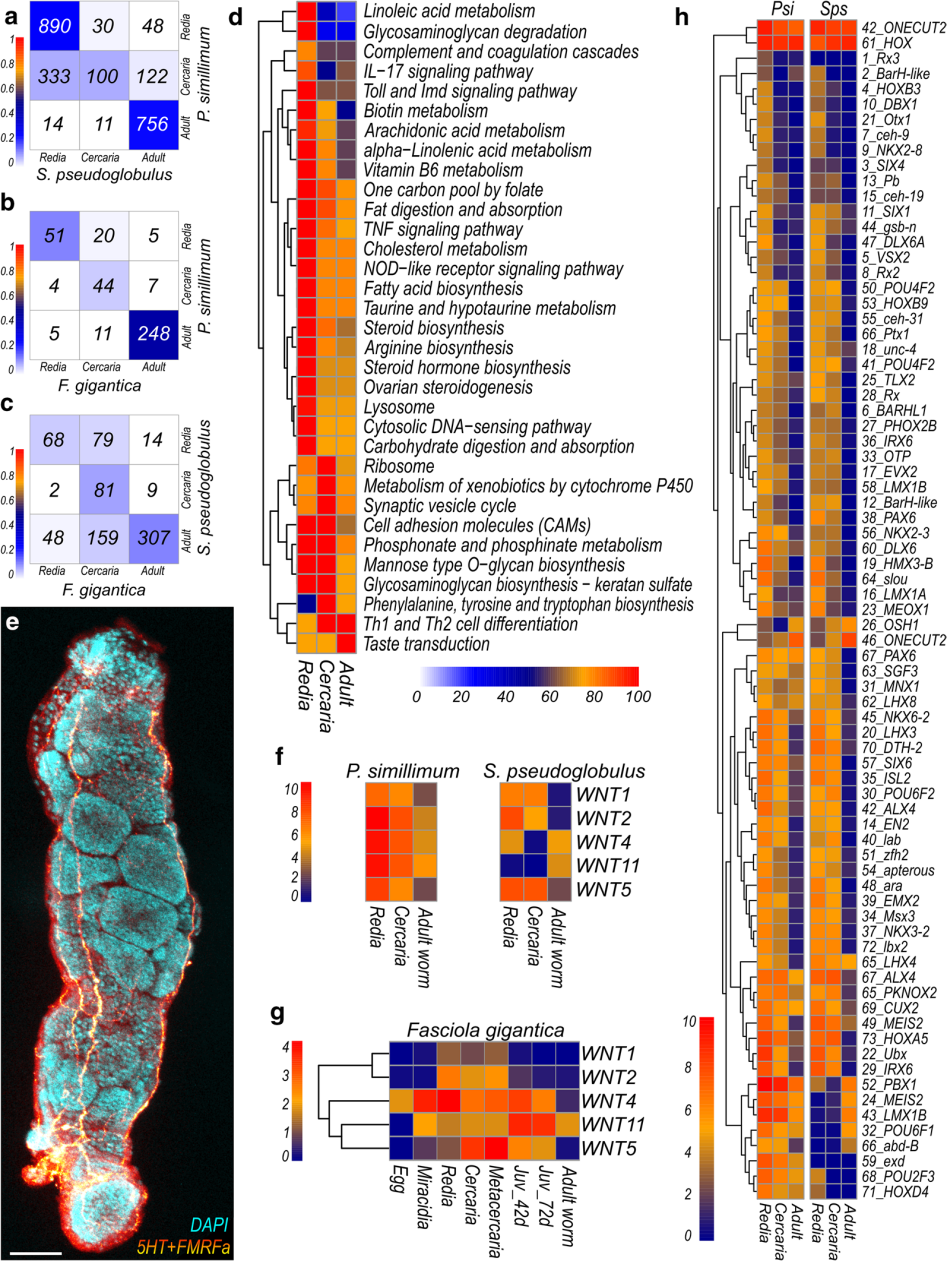
Inter- and intraspecific comparisons of life cycle stages based on the activity of expressed genes. **a**-**c** Comparison of orthologues expression in the redia, cercaria and adult stages of *P. simillimum*, *S. pseudoglobulus* and *F. gigantica*. Color scale represents similarity between stages based on the expression of orthologous genes (Jaccard similarity score). Numbers of orthologous genes with stage-specific expression are shown in each cell of the plots. **d** Activity of metabolic pathways in the redia, cercaria and adult stages of *P. simillimum*. Color scale represents the percent of active enzymes, where maximum number among the stages is taken as 100 percent. Pathways that show more than 20% fluctuation in the number of active enzymes are shown. **e** Confocal microscopy image of *P. simillimum* rediae with developing cercariae embryos. Nuclei are labelled with DAPI, nervous system is labelled with antibodies against 5HT and FMRFamide. **f**, **g** Expression of genes that encode Wnt secreted ligands in three species of Digenea. Color scales represent the log_2_(TPM+1) values. **h** Expression dynamics of the homeobox-containing transcription factors is highly conserved between *P. simillimum* and *S. pseudoglobulus*. Color scale represents the log_2_(TPM+1) values

Parasitic worms evolved from free-living ancestors by intercalation of stages and gradual increase of life cycle complexity [62]. Thus, the comparison of expressed gene sets among the stages within the life cycle as well as between representatives of different species may shed light on the relationships between life cycle stages and the order of their appearance. It is plausible that in inter-specific comparison the most similar patterns of gene expression should be observed between the most ancient life cycle stages and the recently developed stages would differ the most. To address this question, we identified one-to-one orthologs in *P. simillimum*, *S. pseudoglobulus* and *F. gigantica* by reciprocal best BLASTp hits (RBBH), and for each pair of species we compared their life cycle stages in terms of expression dynamics conservation, i.e. compared the sets of stage-specific genes between the species. We found 11,303 RBBH pairs for *P. simillimum* and *S. pseudoglobulus* (Additional file 11: Table S10.1), 6746 RBBH pairs for *P. simillimum* and *F. gigantica* (Additional file 11: Table S10.2), and 7276 RBBH pairs for *S. pseudoglobulus* and *F. gigantica* (Additional file 11: Table S10.3). The comparison of gene expression specificity was performed using Jaccardʼs similarity score. According to the results, between the psilostomatid species analyzed, rediae and adults demonstrated the highest similarity (Jaccard scores = 0.26 and 0.25, respectively) in comparison to free-living cercaria stages (Jaccard score = 0.09) (Fig. 4a). In terms of gene expression, similarity between adults of *P. simillimum* and *F. gigantica* was more noticeable (Jaccard score = 0.45) in contrast to the scores between rediae (Jaccard score = 0.13) and cercariae (Jaccard score = 0.06) stages (Fig. 4b). The scores obtained for rediae, cercariae and adult worm stages of *S. pseudoglobulus* and *F. gigantica* were equal to 0.06, 0.10 and 0.13, respectively (Fig. 4c). Thus, adult worms in *S. pseudoglobulus* and *F. gigantica* expressed more similar sets of genes than cercaria and redia in these species. Similarities between cercaria and redia stages (for example, Fig. 4a, c) might be explained by the presence of developing cercariae inside of the redia stage (see Fig. 4e). As a result, the redia stage transcriptome will inevitably contain transcripts that are active during the embryonic development of the cercaria (see Fig. 4e). In general, less than 16% of one-to-one orthologs showed similar stage-specific expression dynamics between species pairs: 15.4% (1746/11,303) of orthologous genes between *P. simillimum* and *S. pseudoglobulus* (Fig. 4a), 5% (343/6746) between *P. simillimum* and *F. gigantica* (Fig. 4b), and 6.2% (456/7276) between *S. pseudoglobulus* and *F. gigantica* (Fig. 4c).

Another approach to estimate the level of similarity among various life cycle stages was based on the Gene Ontology enrichment analysis results (Additional file 12: Figure S2). Jaccard score applied to the results of GO classification showed, as expected, that all analyzed stages within each species are most similar to themselves (Additional file 12: Figure S2a-f). In *P. simillimum* (Additional file 12: Figure S2b, e), however, there were slight similarities between redia and cercaria stages that can be explained by the presence of developing cercaria individuals inside of a redia stage (Fig. 4e). Similarities between cercaria and adult stages in *S. pseudoglobulus* may be caused by two factors: (i) both stages belong to amphimictic generation of the life cycle; and (ii) the presence of two cryptic species in the samples of *S. pseudoglobulus* (Additional file 12: Figure S2c, f).

In terms of active biological processes and molecular functions, cercaria stages of *P. simillimum* and *S. pseudoglobulus* were more similar to each other than to the parasitic stages (Additional file 12: Figure S2h, k). In a comparison between *P. simillimum* and *F. gigantica*, only adult worms had a Jaccard score above 0.1: 0.16 for sets of “enriched” biological processes (Additional file 12: Figure S2g) and 0.34 for molecular function terms (Additional file 12: Figure S2j). A comparison of Gene Ontology enrichment results between *S. pseudoglobulus* and *F. gigantica* revealed that in terms of biological processes, rediae are more similar to each other than to other stages (Additional file 12: Figure S2i). At the same time, in terms of molecular functions, Jaccard score for cercaria stages of *S. pseudoglobulus* and *F. gigantica* was equal to 0.24, that was higher than the scores obtained for the parasitic stages (Additional file 12: Figure S2l). To summarize, in all comparisons between *P. simillimum*, *S. pseudoglobulus* and *F. gigantica* the number of common biological processes identified for free-living cercaria stage was higher than that obtained for the parasitic stages of the life cycle (Additional file 13: Table S11).

Genes involved in the maintenance and regulation of biological processes must interact throughout the life cycle. Co-expression analysis was performed to identify potentially co-regulated groups of genes based on similarities of their expression dynamics. For both analyzed psilostomatid species 12 co-expressed clusters were identified (Additional file 14: Figure S3). In *P. simillimum* and *S. pseudoglobulus* 12,708 and 27,919 genes were included into the clusters which comprises approximately 59 and 60 percent of the total number of analyzed genes, respectively. As shown in Additional file 14, cluster size varies from 417 to 2890 genes for *P. simillimum* and from 55 to 5910 for *S. pseudoglobulus*. Results of the GO-enrichment analysis for each cluster of co-expressed genes are shown in Additional file 15: Table S12 and Additional file 16: Table S13. For instance, genes that are significantly upregulated in parasitic stages (cluster C4 in *P. simillimum*, Additional file 14: Figure S3a) are enriched for GO-terms associated with cell cycle, meiosis, and reproduction (Additional file 15: Table S12.5). At the same time, high activity of genes involved in the function of the nervous system is clearly seen in the cercaria stage (see cluster 10 in Additional file 14: Figure S3a, Additional file 15: Table S12.11).

As a next step we analyzed the activity of metabolic pathways in the redia, cercaria and adult worm stages of *P. simillimum.* Analysis in *S. pseudoglobulus* has not been performed due to the presence of cryptic species. The number of active enzymes belonging to each pathway was calculated for each stage (Additional file 17: Table S14). Pathways demonstrating more than 20% fluctuation in the number of active enzymes between stages are shown in Fig. 4d. The majority of pathways with potential stage-specific regulation (23/33) are stronger activated in the redia stage, whereas only four pathways in cercaria (“Ribosome”, “Metabolism of xenobiotics by cytochrome P450”, “Synaptic vesicle cycle”, and “Phenylalanine, tyrosine and tryptophan biosynthesis”) and one in the adult worm stage (“Taste transduction”) demonstrate higher activity.

It is important to note that parasites receive nutrients from the host and, for example, the biosynthesis of fatty acids in digeneans is known to be reduced. According to a previous publication [13], only three enzymes in the fatty acid biosynthesis pathway were identified in genomes of *C. sinensis*, *S. mansoni* and *S. japonicum*, namely acetyl-CoA carboxylase (EC: 6.4.1.2, 6.3.4.14), FabD (EC: 2.3.1.39) and FabF (EC: 2.3.1.179). Surprisingly, in addition to three aforementioned enzymes, we identified 1 protein with code 2.3.1.85 and 11 enzymes with code 6.2.1.3 in the reference gene set for *P. simillimum*. The first one has high similarity to FasN, but contains only one key domain, “Acyl_transf_1”, suggesting this sequence to be probably not a full orthologue of FasN. Eleven proteins with code 6.2.1.3 match long-chain-fatty-acid-CoA ligase. This enzyme activates long-chain fatty acids both for the synthesis of cellular lipids as well as for their degradation *via* beta-oxidation. It seems, that similar to that in other flukes, the fatty acid biosynthesis pathway is reduced in *P. simillimum*, reflecting the necessity to receive lipids from its host, but contains an additional component that is missing in several other species.

### Activity of Wnt and Homeobox genes

Wnt ligands are known to be dynamically expressed throughout life cycles which corresponds well to their important function in the morphogenetic processes [63]. Compared to free-living bilaterians, parasitic worms have reduced sets of Wnt ligands and homeobox containing transcription factors [9, 10, 64]. In accordance with the previous reports only five secreted ligands of the Wnt pathway (*Wnt*-1, 2, 4, 5, 11) were identified in the transcriptomes of *P. simillimum* and *S. pseudoglobulus.* Interestingly, in both species the expression of *Wnt-1*, *Wnt-2* and *Wnt-5* genes was stronger in the redia and cercaria stages than in the adult worms (Fig. 4f). Similar expression dynamics was observed in *F. gigantica* where the expression of *Wnt-1* and *Wnt-2* is restricted to the larval stages and only *Wnt-11* is strongly expressed in the adult worms (Fig. 4g).

Homeobox genes and homeodomain containing proteins function as transcription factors that activate or repress gene expression [65]. Transcription factors play a key role in decoding the genetic blueprint and converting it through a cascade of events into cell fate decisions and cell differentiation that ultimately gives rise to a complex multicellular organism [65]. In *P. simillimum* and *S. pseudoglobulus* we identified 77 and 75 transcription factors with homeobox domains, respectively (Fig. 4h). Two genes, *Rx3* and *Exd* were not detected in *S. pseudoglobulus* transcriptome but were present in *P. simillimum.* As shown in Fig. 4h, the majority of homeobox genes are upregulated in the redia and cercaria stages. Another interesting observation is the high degree of congruence between the expression patterns of homeobox-containing genes in *P. simillimum* and *S. pseudoglobulus*. While the overall level of gene expression congruence between these species is ~ 15% (see Fig. 4a), the expression of transcription factors is highly conserved and clearly stand out.

### Differential expression of cathepsins and aquaporins

**Fig. 5:**
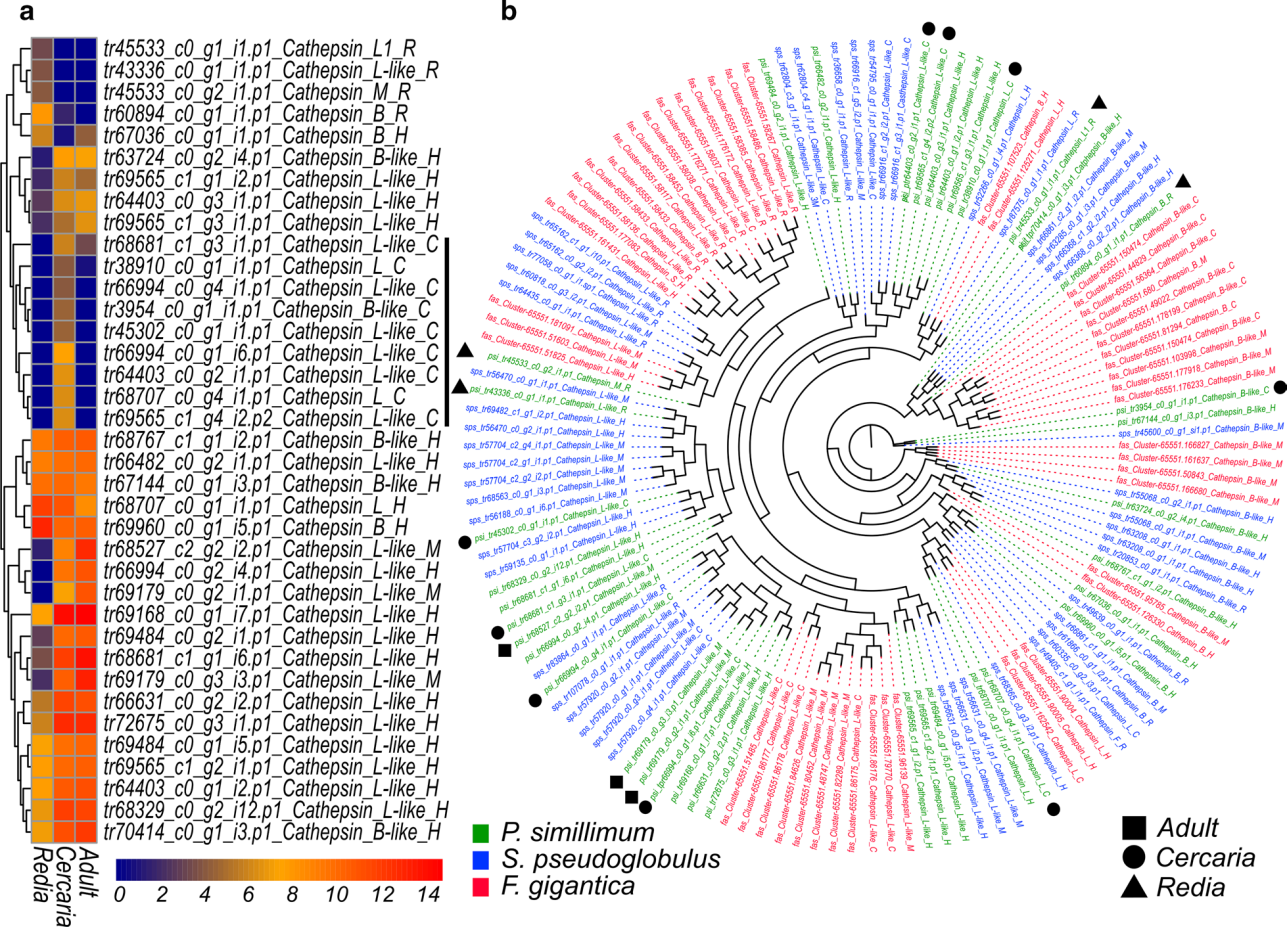
Expression and evolution of cathepsin gene family. **a** Expression dynamics of 37 cathepsin genes of *P. simillimum*. Color scale represents the log_2_(TPM+1) values. **b** Phylogenetic relation of cathepsins in *P. simillimum* (green), *S. pseudoglobulus* (blue) and *F. gigantica* (red). Maximum likelihood tree. The geometric shapes label the sequences with stage-specific expression in redia (triangle), cercaria (circle) or adult worm (box) of *P. simillimum*. *Abbreviations*: R, redia-specific; C, cercaria-specific; M, adult-specific; H, with ubiquitous expression

Consistent with the previous reports in fasciolids [25] and other digeneans [28, 29, 31, 34, 35], large groups of proteases belonging to cathepsin L and cathepsin B families were identified in the Psilostomatidae. In *P. simillimum*, 37 genes encoding cathepsins are present (Fig. 5a). Similar to *F. gigantica*, 16 of 37 genes are developmentally regulated with four genes having preferential expression in the redia, nine in the cercaria and four in the adult stage. Interestingly, in contrast to *F. gigantica*, five cathepsin B-like peptidases have strong expression in the redia stage while the majority of redia-specific cathepsins in *F. gigantica* belong to the L group. The phylogenetic analysis indicated that several families of cathepsins, especially those with cercaria-specific expression, do not have orthologs in *F. gigantica* and most probably were expanded in the psilostomatid lineage (Fig. 5b).

**Fig. 6:**
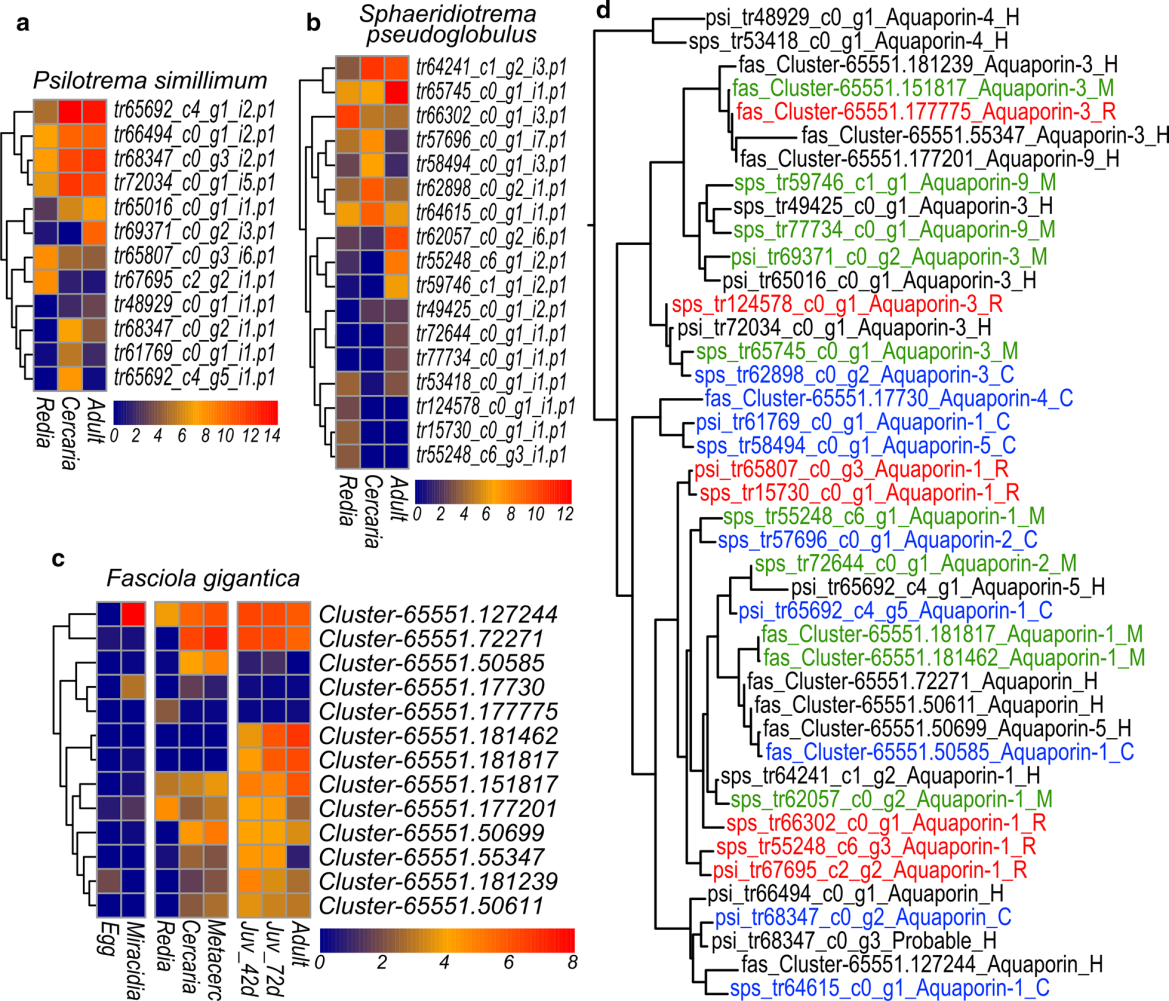
Expression and evolution of aquaporins. **a**-**c** Expression dynamics of aquaporins in *P. simillimum*, *S. pseudoglobulus* and *F. gigantica*. Color scales represent the log_2_(TPM+1) values. **d** Maximum likelihood tree depicting the phylogenetic relation of aquaporins in *P. simillimum*, *S. pseudoglobulus* and *F. gigantica*. Stage-specific proteins are labeled with green (adults), blue (cercaria) and red (redia)

Another interesting result which to some extend parallels the observations with cathepsins is the differential expression of aquaporin genes (Fig. 6a–c). These proteins are important for the transport of water and small molecules through the cellular membrane as well as for the maintenance of proper osmotic pressure in tissues. Aquaporin gene families consist of 12 genes in *P. simillimum*, 17 genes in *S. pseudoglobulus* and 13 genes in *F. gigantica*. As shown in Fig. 6a–c, aquaporins are differentially regulated during the life cycle and genes with strict expression in the redia, cercaria or adult worm. Larger number of aquaporins in *S. pseudoglobulus* might be caused by the presence of a cryptic species, but in *P. simillimum* as well as in *F. gigantica* lineage-specific expansions of the gene families are obvious (Fig. 6d).

## Discussion

Life cycles of digeneans are based on the alternation of parthenogenetic and amphimictic generations. Each generation has several stages with own ontogeny and disparate morphological and physiological traits. Differential gene expression is the major mechanism which creates such a remarkable diversity within a life history. One of the approaches to estimate the similarities and differences among the stages and between different species is a comparative transcriptomic analysis. Given the high diversity of complex life cycles of the Digenea, preferable models to uncover the molecular basis of life cycle regulation are the species that combine evolutionary primitive and advanced features. Thus, the members of the family Psilostomatidae can be regarded as a good choice for comparative studies which is additionally facilitated by the close phylogenetic relationships between *Psilotrema simillimum* and *Sphaeridiotrema pseudoglobulus*.

Quality and completeness of the gene sets obtained for *P. simillimum* and *S. pseudoglobulus* are comparable to that in other species of Digenea (Fig. 2a) and, therefore, can serve as reliable references for further studies. Unfortunately, the direct comparison of expression dynamics between two species is complicated by the presence of the cryptic species in *Sphaeridiotrema*. Our assumption for the existence of two cryptic species is based on the approximately 2-fold difference in gene number between *P. simillimum* and *S. pseudoglobulus* datasets, unusually high level of duplicated orthologues (Fig. 2a), and the presence of two different haplotypes of *cox*1 in the transcriptome of *Sphaeridiotrema* (Additional file 6: Figure S1). According to the results of expression analysis (Fig. 3b, d), the samples from rediae and adult worms contained a mixture of individuals from two cryptic species while cercaria samples contained exclusively *S. pseudoglobulus* individuals. The presence of the second species has a direct impact on the identification of orthologous genes and differential genes expression analysis. In the first case, the influence is noticeable in the relatively high number of species-specific orthologous groups (OGs) and proteins included into them (43 OGs with 435 proteins in *S. pseudoglobulus*) (Fig. 2c, Additional file 7: Table S6.4). In the second case, a large number of genes with preferential expression is observed in the redia and adult stages (see Fig. 3d). Comparison of expression dynamics between *P. simillimum* and *S. pseudoglobulus*, however, is not compromised because it is based on the identification of one-to-one reciprocal best BLASTp hits (RBBH). Nevertheless, we assume that our conclusions about differential gene expression and the absolute numbers of upregulated and downregulated genes are more reliable for *P. simillimum* than for *S. pseudoglobulus*. Interestingly, BUSCO analysis of the recently published transcriptome of *F. gigantica* [25] also detects an elevated number of duplicated proteins (53%) (Fig. 2a). Moreover, the proportion of genes with the specific expression in the cercaria stage (32.18%) is much higher than that in the redia (5.29%) and adult (9.08%) stages (Fig. 3e). These observations resemble the situation in the redia and adult worm stages of *S. pseudoglobulus* albeit the presence of any cryptic species in *F. gigantica* has not been reported.

How is the common genetic information utilized at different stages of a life cycle? It is plausible that the degree of overlap in the sets of active genes among the stages is correlated with the level of similarity in their physiology and anatomy. The larger are the differences the smaller should be the overlap between the sets of active genes among the stages. Each stage of a life cycle needs certain number of housekeeping genes, signaling molecules and ubiquitous transcription factors that are indispensable and, therefore, must be expressed by all the stages. However, development of highly specialized stage-specific morphological structures and adaptations to various environments require dedicated groups of genes. Theoretically, these specialized genes can be expressed exclusively in just one stage (i.e. “stage-specific genes”) or get activated in two or more stages of a life cycle.

According to the results of the overlap analysis (Fig. 3a, b), we can identify three groups of genes based on their expression dynamics. The first group contains genes active in all analyzed life cycle stages. The second group includes sequences with expression in two different stages of the life cycle only. The last group contains genes with exclusive expression in only one life cycle stage. The proportions of sequences assigned to these three groups vary significantly between *P. simillimum* (Fig. 3a) and *S. pseudoglobulus* (Fig. 3b). In *P. simillimum* more than 60% of analyzed sequences are active in the redia, cercaria, and adult worm stages, while in *S. pseudoglobulus* less than 40% of genes exhibit such an expression pattern. The presence of a species complex in the sample of *S. pseudoglobulus* is probably one of the reasons for the above-mentioned difference between the analyzed species. For example, simultaneous presence of orthologous genes from two cryptic species in the redia and adult stages, which is not observed in cercaria, results in underestimated number of genes that are active in all three stages (see Fig. 3d).

Parthenogenetic and amphimictic generations have both the free-living and parasitic stages. Each stage of a life cycle exists in a specific environment and needs numerous adaptations in order to survive. From a molecular point of view, the completion of a life cycle is impossible without a differential gene expression. In this respect it is important to know how many genes are expressed in a strict stage-specific manner. In *P. simillimum* (Fig. 3c), *F. gigantica* (Fig. 3e), and *S. pseudoglobulus* (Fig. 3d), 7805 (36.41%), 31,395 (46.54%), and 30,622 (65.96%) genes demonstrate strict stage-specific expression dynamics, respectively. It is noticeable that even when using a very strict criterion (80% of total expression of a gene must be restricted to just one stage of the life cycle), the considerable proportion of sequences belongs to this “stage-specific” category. With any decrease of the 80% threshold used, the absolute number and proportion of genes with stage-specific expression will only increase.

Despite the absence of a common trend in “partitioning” of the genomes according to the life cycle stages, the results obtained in *P. simillimum* and *S. pseudoglobulus* have three important implications. First, the genetic network responsible for the development of the stage-specific traits seems to be complex and includes genes with different modes of expression dynamics. Second, the proportion of “stage-specific” genes, i.e. genes with exclusive expression in only one stage, is relatively small (for example, 7.12–16.36% in *P. simillimum*). Similar trends were observed in other organisms with complex life cycles such as cestodes and medusozoan cnidarians [63, 64]. Third, all stages in a life cycle are “coupled” by thousands of genes, that are active at several stages.

Changes in gene expression reflect functional specializations of the stages within a life cycle which can be identified by Gene Onthology (GO) analysis of the stage-specific genes. The major role of the parasitic stages is to increase the number of new individuals at the expense of the host resources. That function is consistent with the enrichment of biological processes such as “Positive regulation of embryonic development” (Additional file 9: Table S8.1), “Female meiotic nuclear division” (Additional file 9: Table S8.4, 7), “Determination of left/right symmetry” (Additional file 9: Table 8.3, 6, 9) in the rediae and adult worms of *P. simillimum* (Additional file 9: Table S8.1, 3), *S. pseudoglobulus* (Additional file 9: Table S8.4, 6), and *F. gigantica* (Additional file 9: Table S8.7, 9). Considerable upregulation of Wnt (Fig. 4f, g) and homeobox-containing genes (Fig. 4h) clearly reflects active developmental processes (cercaria embryogenesis) that take place inside the rediae. Free-living larvae (cercariae) are known to exhibit complex behavioral activities and that is reflected by the enrichment of biological processes associated with the nervous system (Additional file 15: Table S12.11) and muscular locomotion (Additional file 9: Table S8.2, 5). Taken together, the results of the transcriptomic analysis demonstrate a robust correlation between gene expression and corresponding biological processes that underlay well-known stage-specific functions.

Given the overlaps in the sets of expressed genes between stages analyzed, the successful completion of a digenean life cycle requires complex system to regulate the activity of a genome. As evident from our co-expression analysis, there are at least 12 complex networks where differentially expressed genes can be located in central nodes, intermediate ones, and in the periphery (Additional file 14: Figure S3). Regardless of a topology, a genetic network functions *via* coordinated upregulation and downregulation of large groups of genes. According to the results of co-expression analysis, the majority of the *P. simillimum* and *S. pseudoglobulus* genes (59 and 60 percent, respectively) change their expression during the life cycle according to just twelve expression profiles (Additional file 14: Figure S3). In our analysis, we only focused on the protein-coding genes with a relatively high levels of expression (≥ 1 TPM) as the interaction between the gene products are usually the main source of species-, generation- and stage-specific traits. At the same time, large proportion of the assembled transcripts with lengths > 200 nucleotides did not have open reading frames. While some of them surely represent assembly artifacts, others might be functionally relevant. According to the recent publications [66–71] long non-coding RNAs (ncRNAs) are one of the important regulators of gene expression and indispensable components of genetic networks. Analysis of the diversity and roles of non-coding RNAs in the Psilostomatidae is, therefore, a promising research direction for the future.

Our results demonstrate that during the life cycle, many genes change their expression significantly. As a result, each stage has a molecular signature that is defined by the unique combination of active genes as well as their stage-specific expression levels. Stage-specific changes in gene expression are well illustrated by the cathepsins (Fig. 5) and aquaporins (Fig. 6) that have a potential role in adaptation to contrasting environmental conditions associated with different hosts. Similar trend is also noticeable, for example, in the number of active enzymes associated with various molecular processes (Fig. 4d). For example, the larger number of active enzymes in the synaptic vesicle cycle in the cercaria (Fig. 4d) is possibly connected with the environment scanning performed by the larva while it searches the best place for encystment. Given that the embryonic development is an energy- and resource-consuming process, high activity of various biosynthetic processes in the redia stage is expected (Fig. 4d). Differences between the stages, therefore, can be well explained according to the biological characteristics of each stage.

Comparison of different digenean species is interesting not only for analyzing the similarities and differences in their biology but also for understanding the evolution of complex life cycles. Coupling between the stages of a cycle in terms of shared genes should impose limits on how individual genes may evolve and their expression may change. From this point of view, the main question is how did the different stages of the complex life cycles evolve? Two possible scenarios may be considered. According to the first one, “the hypothesis of coupled evolution”, we would expect that almost the same sets of genes are expressed in different stages of a life cycle [72]. Under this model, any mutations in these genes would be mostly maladaptive, since it is unlikely that such changes would be simultaneously favored by selection in different stages [72]. In another scenario, referred to as “the hypothesis of uncoupled evolution”, morphological traits in each stage are encoded by different sets of genes [72]. In this case the selection process may act independently in each stage of the life cycle [72]. According to our results, the sets of genes associated with each stage largely overlap, but also differ from each other. At the same time, the noticeable number of genes involved in stage-specific signatures are simultaneously active at two stages of the life cycle (Fig.3 a, b). Thus, both aforementioned scenarios appear to coexist and one of the topics for further research should be a more detailed analysis of the molecular regulation of complex life cycles and the relationship of regulatory mechanisms between the stages.

In our study, we carried out a comparative transcriptomic analysis of two psilostomatid species, and also compared both species with the data from *F. gigantica*. A comparison between the species should reveal how the molecular signatures of homologous life cycle stages change in evolution. In terms of active gene sets (expression dynamics of orthologs in two species), parasitic stages of the *P. simillimum* and *S. pseudoglobulus* are more similar, than free-living stages (Fig. 4a). Among both species of the Psilostomatidae and *F. gigantica*, the number of one-to-one orthologs with similar expression pattern is higher in adult worms than between any other stages (Fig. 4b, c). We hypothesize that this result may be related to the evolutionary origin of complex life cycles: expression dynamics and expressed gene sets are more conserved between the most ancient stages, while recently evolved stages are more variable. From this point of view, similar patterns of orthologues expression should be observed in closely related species living in similar conditions, but the proportion of such genes may vary from stage to stage. Surprisingly, less than 16% of one-to-one orthologues demonstrate similar expression dynamics throughout a life cycle (Fig. 4a–c). The observed degree of similarities is lower than to be expected from close phylogenetic relationship of *P. simillimum* and *S. pseudoglobulus*. Thus, the analysis of gene expression dynamics reveals a high level of plasticity in transcriptional regulation among digenean species.

When discussing both conservative and variable components of the stage-specific expression signatures, it should be noted that the transcriptome is a snapshot of the genome activity at a certain moment and it is not a compulsory representation of all expression changes in a life history. For example, cercariae undergo embryonic development inside the redia stage, have a free-living period, and ultimately become metacercariae/adolescariae. In our study, transcriptomes from two periods of the cercarial ontogeny were obtained. The first one is a transcriptome of cercaria embryos that is a part of the rediae transcriptome. The second one is a transcriptome of the free-living cercaria that just have left the first intermediate host. Transcriptomic signal from cercarial embryos developing in the redia stage is of particular interest because here we can expect the genetic and developmental programs to switch from redia to cercaria stage. Our present data set is restricted to just three time points, but we think that the similarity in expression dynamics of homeobox-containing genes during embryogenesis (Fig. 4h) and variations in orthologues expression during life cycles is consistent with the extended hourglass model of development [73]. In a broader context, the hourglass model is used to speculate about the origin and evolution of extant body plans [74]. Extension of this model to complete life span of an animal suggests that a period of high conservation in morphology and gene expression is usually associated with embryogenesis and it is not typical for post-embryonic development [74]. Drost et al. [74] hypothesize that an additional period of conservation may exist among the species that have a metamorphosis steps in their life cycles. Hence, in the digenean complex life cycles with alternating generations and multiple ontogenies, we can expect at least five periods where expression dynamics might be highly conserved among the species. The first three periods are associated with embryonic development of miracidia, rediae (or daughter sporocysts) and cercariae. Two additional phylotypic stages might coincide with the metamorphosis of miracidium into mother sporocyst and transition from cercaria to metacercaria (or adolescaria). Rediae and daughter sporocysts develop directly, without metamorphosis, but we expect that the molecular pathways that regulate these transitions might be also highly conserved across the species. Considering the multiple self-reproduction cycles of parthenogenetic generation individuals and the fact that rediae and daughter sporocysts also produce larvae of the amphimictic generation, a complex regulatory system must be present. Genetic switching between molecular programs of different stages need to take place at these transition points. We suggest that the molecular basis of such switching, as well as coordination of stage-specific traits formation, constitute the most conservative modules of complex life cycle regulation.

Benesh [75] previously reviewed the relationships between different stages in parasitic life cycles and proposed that there is evidence for both autonomy as well as integration. Our results agree with this observation. In addition to ubiquitous housekeeping genes shared by all stages, each stage of a life cycle expresses unique stage-specific genes and there are genes specifically upregulated in more than one stage. Thus, both “uncoupled” and “coupled” evolutionary scenarios take place. We also agree with Benesh [75] in conclusion, that holistic thinking about parasites with complex life cycles is needed because “stages may be linked through genetics (all stages share a genome), ontogeny (larval tissues become those of adults), selection (beneficial changes in one stage can favor correlated changes in others) and the environment (carryover and parental effects)”. The data obtained in our study, we hope, will serve as useful resource in further studies of complex life cycle regulation and evolution.

## Conclusions

During the life cycles most of the genes change their expression levels considerably, consequently the molecular signature of a stage is not only a unique set of expressed genes, but also the specific levels of their expression. Our results indicate unexpectedly high level of plasticity in gene regulation between closely related species. Transcriptomes of *P. simillimum* and *S. pseudoglobulus* provide high quality reference resource for future evolutionary studies and comparative analyses.

## Supplementary information

**Additional file 1: Table S1.** Summary of library preparation

**Additional file 2: Table S2.** Quality and completeness statistics for the transcriptomes.

**Additional file 3: Table S3**. The mapping rates for RNAseq libraries.

**Additional file 4: Table S4.** BUSCO results for publicly available data for the Digenea.

**Additional file 5: Table S5**. Status of orthologues from Metazoa-odb9 in digenean species analyzed.

**Additional file 6: Figure S1.** The phylogenetic tree for *S. pseudoglobulus* and closely related digenean species based on the analysis of cytochrome *c *oxidase subunit 1 (*cox*1) sequences. IDs of two identified *cox*1 sequences from *S. pseudoglobulus* are labeled in red. The transcriptome of *S. pseudoglobulus* contains the *cox*1 sequence from the second, not yet described cryptic species closely related to *S. pseudoglobulus.*

**Additional file 7: Table S6**. Summary of orthologues identification.

**Additional file 8: Table S7.** Annotations of proteins included into Psilostomatidae-specific orthogroups.

**Additional file 9: Table S8.** Results of Gene Ontology enrichment analysis for the life cycle stages (Biological processes).

**Additional file 10: Table S9.** Results of Gene Ontology enrichment analysis for the life cycle stages (Molecular functions).

**Additional file 11: Table S10.** Reciprocal Best BLAST hits (RBBH) between analyzed species.

**Additional file 12: Figure S2.** Inter- and intra-specific comparisons of life cycle stages based on the sets of “enriched” Gene Ontology (GO) terms. Color scale represents the level of similarity between stages (Jaccard similarity score). **a**–**f** Intraspecific comparison between redia, cercaria and adult worm stages for *F. gigantica *(**a**, **d**), *P. simillimum *(**b**, **e**) and *S. pseudoglobulus* (**c**, **f**). Interspecific comparison between redia, cercaria and adult worm stages of *F. gigantica* and *P. simillimum* (**g**, **j**), *S. pseudoglobulus* and *P. simillimum* (**h**, **k**), *F. gigantica* and *S. pseudoglobulus *(**i**, **l**). Analysis was performed on the sets of “enriched” GO-terms describing biological processes (**a**, **b**, **c**, **g**, **h**, **i**) and molecular functions (**d**, **e**, **f**, **j**, **k**, **l**).

**Additional file 13: Table S11**. The common “enriched” GO-terms (Biological processes) for the life cycles stages in the pairs of analyzed species.

**Additional file 14: Figure S3.** Clusters of co-expressed genes. The vertical axis shows the normalized expression values, the horizontal axis shows the life cycles stages (R, redia; C, cercaria; M, adult worm) of *P. simillimum* (**a**) and *S. pseudoglobulus *(**b**).

**Additional file 15: Table S12.** Results of Gene Ontology enrichment analysis for co-expression clusters (Biological processes).

**Additional file 16: Table S13.** Results of Gene Ontology enrichment analysis for co-expression clusters (Molecular functions).

**Additional file 17: Table S14. **Number of active enzymes in analyzed life cycle stages of *P. simillimum.*

## Data Availability

Data supporting the conclusions of this article are included within the article and its additional file. BioProject has been deposited at NCBI under accession PRJNA516017. Summary tables with all obtained results of transcriptomic analysis of both Psilostomatidae species are available from figshare: (i) *Psilotrema simillimum*: https://doi.org/10.6084/m9.figshare.12410069.v1, (ii) *Sphaeridiotrema pseudoglobulus*: https://doi.org/10.6084/m9.figshare.12410225.v1.

## Abbreviations

TPM: Transcripts-per-million
NCBInt: The nucleotide database of the National Center for Biotechnology Information
NCBInr: The non-redundant protein database of the National Center for Biotechnology Information
BUSCO: Benchmarking universal single-copy orthologs
*cox*1: Cytochrome *c* oxidase subunit 1
OG: Orthogroups
ABC transporter: The ATP-binding cassette transporter
GO: Gene Ontology
BLAST: Basic Local Alignment Search Tool
RBBH: Reciprocal best BLASTp hits
EC: The enzyme commission number
HMM: Hidden Markov model
KEGG: Kyoto Encyclopedia of Genes and Genomes
PBS: Phosphate-buffered saline
SCB: Sodium cacodylate buffer
