# Supplementary figures and images for: Molecular signatures of the rediae, cercariae and adult stages in the complex life cycles of parasitic flatworms (Digenea: Psilostomatidae)
Source: Parasit Vectors. 2020 Nov 10;13:559. doi: 10.1186/s13071-020-04424-4 (PMC7653818; doi:10.1186/s13071-020-04424-4)

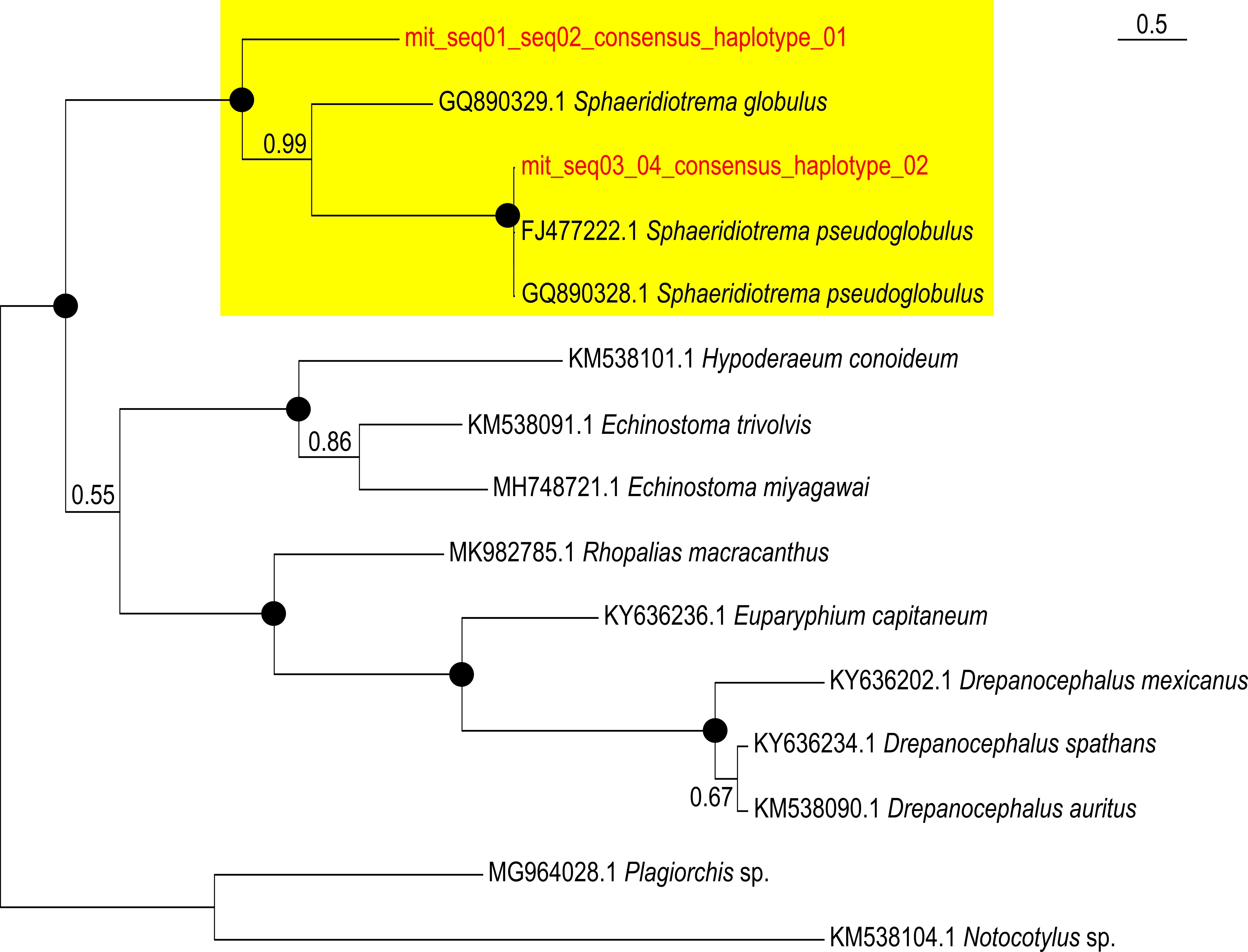

Supplement: Supplementary file 6 — Additional file 6: Figure S1. The phylogenetic tree for S. pseudoglobulus and closely related digenean species based on the analysis of cytochrome c oxidase subunit 1 (cox1) sequences. IDs of two identified cox1 sequences from S. pseudoglobulus are labeled in red. The transcriptome of S. pseudoglobulus contains the cox1 sequence from the second, not yet described cryptic species closely related to S. pseudoglobulus. [file 13071_2020_4424_MOESM6_ESM.png]

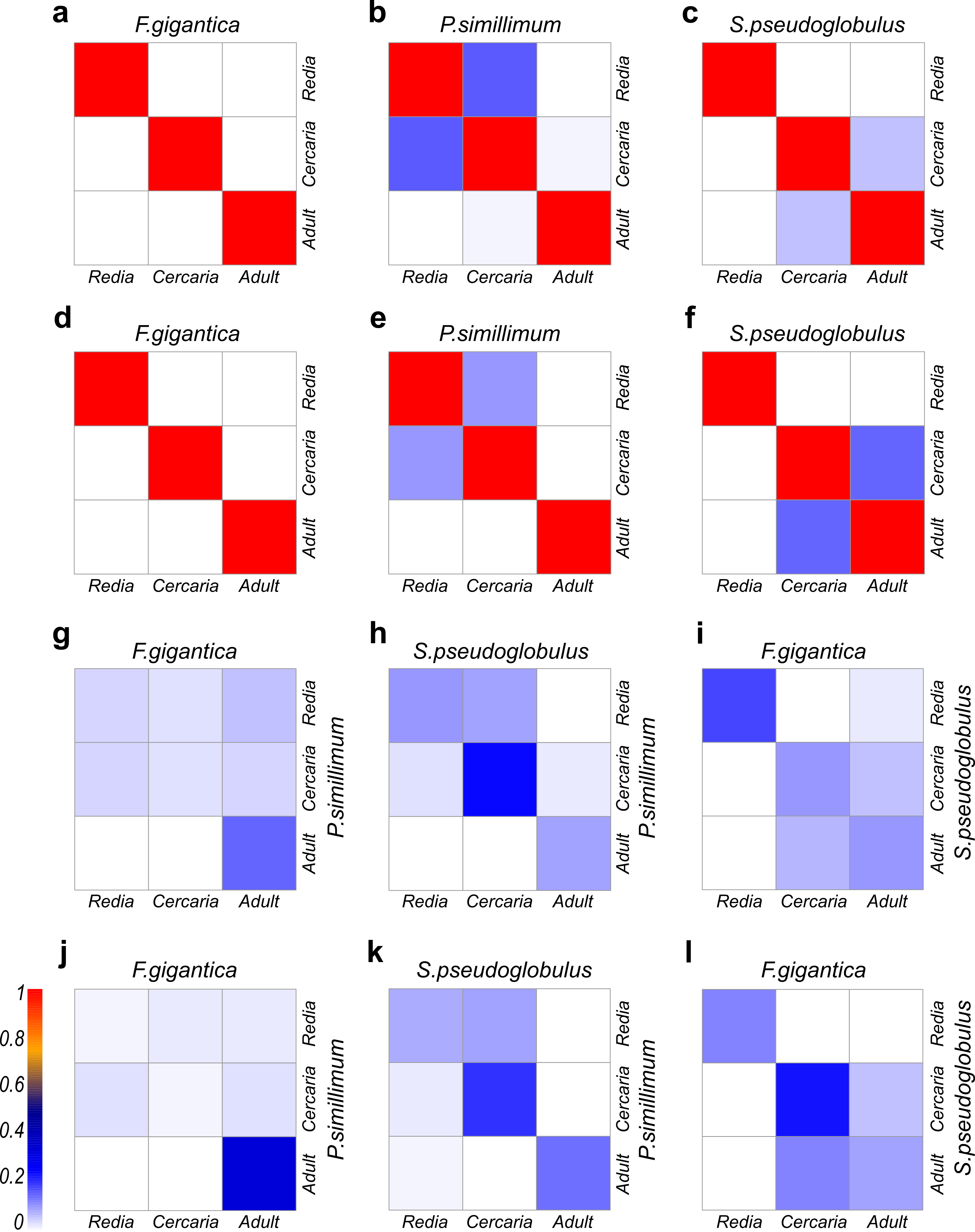

Supplement: Supplementary file 12 — Additional file 12: Figure S2. Inter- and intra-specific comparisons of life cycle stages based on the sets of “enriched” Gene Ontology (GO) terms. Color scale represents the level of similarity between stages (Jaccard similarity score). a–f Intraspecific comparison between redia, cercaria and adult worm stages for F. gigantica (a, d), P. simillimum (b, e) and S. pseudoglobulus (c, f). Interspecific comparison between redia, cercaria and adult worm stages of F. gigantica and P. simillimum (g, j), S. pseudoglobulus and P. simillimum (h, k), F. gigantica and S. pseudoglobulus (i, l). Analysis was performed on the sets of “enriched” GO-terms describing biological processes (a, b, c, g, h, i) and molecular functions (d, e, f, j, k, l). [file 13071_2020_4424_MOESM12_ESM.png]

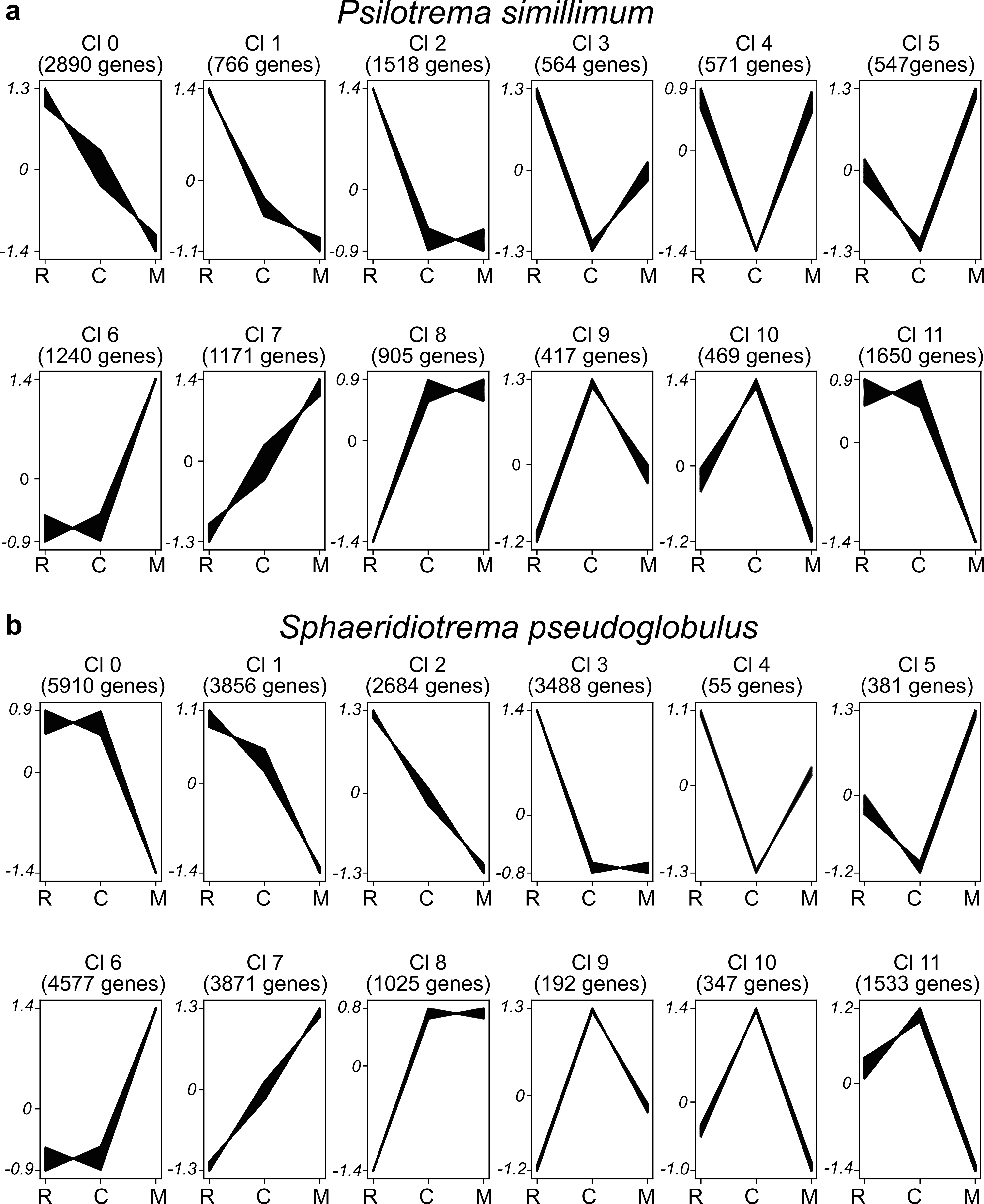

Supplement: Supplementary file 14 — Additional file 14: Figure S3. Clusters of co-expressed genes. The vertical axis shows the normalized expression values, the horizontal axis shows the life cycles stages (R, redia; C, cercaria; M, adult worm) of P. simillimum (a) and S. pseudoglobulus (b). [file 13071_2020_4424_MOESM14_ESM.png]
